# Loneliness, traditional risk factor control, genetic predisposition, and development of musculoskeletal disorders

**DOI:** 10.1093/rheumatology/keag326

**Published:** 2026-06-26

**Authors:** Xiaofeng Ma, Xin Song, Yanqiu Zou, Di Zhang, Bin Yang, Bowen Lei, Jinyu Zhou, Xunying Zhao, Rong Xiang, Yang Qu, Sirui Zheng, Ting Yu, Tao Han, Yangdan Zhong, Maoyao Xia, Mengyu Fan, Xia Jiang, Ben Zhang

**Affiliations:** Department of Epidemiology and Biostatistics, West China School of Public Health and West China Fourth Hospital, Sichuan University, Chengdu, Sichuan, China; Department of Epidemiology and Biostatistics, West China School of Public Health and West China Fourth Hospital, Sichuan University, Chengdu, Sichuan, China; Department of Epidemiology and Biostatistics, West China School of Public Health and West China Fourth Hospital, Sichuan University, Chengdu, Sichuan, China; Department of Epidemiology and Biostatistics, West China School of Public Health and West China Fourth Hospital, Sichuan University, Chengdu, Sichuan, China; Department of Nutrition and Food Hygiene, West China School of Public Health and West China Fourth Hospital, Sichuan University, Chengdu, Sichuan, China; Department of Epidemiology and Biostatistics, West China School of Public Health and West China Fourth Hospital, Sichuan University, Chengdu, Sichuan, China; Department of Nutrition and Food Hygiene, West China School of Public Health and West China Fourth Hospital, Sichuan University, Chengdu, Sichuan, China; Department of Epidemiology and Biostatistics, West China School of Public Health and West China Fourth Hospital, Sichuan University, Chengdu, Sichuan, China; Department of Nutrition and Food Hygiene, West China School of Public Health and West China Fourth Hospital, Sichuan University, Chengdu, Sichuan, China; Department of Epidemiology and Biostatistics, West China School of Public Health and West China Fourth Hospital, Sichuan University, Chengdu, Sichuan, China; Department of Nutrition and Food Hygiene, West China School of Public Health and West China Fourth Hospital, Sichuan University, Chengdu, Sichuan, China; Department of Epidemiology and Biostatistics, West China School of Public Health and West China Fourth Hospital, Sichuan University, Chengdu, Sichuan, China; Department of Nutrition and Food Hygiene, West China School of Public Health and West China Fourth Hospital, Sichuan University, Chengdu, Sichuan, China; Department of Nutrition and Food Hygiene, West China School of Public Health and West China Fourth Hospital, Sichuan University, Chengdu, Sichuan, China; Department of Nutrition and Food Hygiene, West China School of Public Health and West China Fourth Hospital, Sichuan University, Chengdu, Sichuan, China; Department of Epidemiology and Biostatistics, West China School of Public Health and West China Fourth Hospital, Sichuan University, Chengdu, Sichuan, China; Department of Epidemiology and Biostatistics, West China School of Public Health and West China Fourth Hospital, Sichuan University, Chengdu, Sichuan, China; Department of Nutrition and Food Hygiene, West China School of Public Health and West China Fourth Hospital, Sichuan University, Chengdu, Sichuan, China; Department of Clinical Neuroscience, Karolinska Institute, Stockholm, Sweden; Department of Epidemiology and Biostatistics, West China School of Public Health and West China Fourth Hospital, Sichuan University, Chengdu, Sichuan, China; Hainan General Hospital and Hainan Affiliated Hospital, Hainan Medical University, Haikou, China

**Keywords:** loneliness, social isolation, musculoskeletal disorders, cohort study

## Abstract

**Objectives:**

The objectives of this study were to investigate prospective associations of loneliness and social isolation with musculoskeletal (MSK) disorder risk, to evaluate their relative importance relative to that of traditional risk factors, and assess interactions with genetic predispositions.

**Methods:**

We analysed data from 315 197 UK Biobank participants. Loneliness and social isolation were assessed through self-reported questionnaires. The primary outcome was the incidence of any MSK disorder, with secondary outcomes comprising five specific MSK conditions of high burden [osteoarthritis, gout, rheumatoid arthritis, low back pain, and neck pain]. Cox regressions were performed to examine the associations between loneliness, social isolation, traditional risk factor control, polygenic risk score, and risk of overall and specific MSK disorders.

**Results:**

During a median follow-up of 13.3 years, 79 689 MSK cases were documented. Loneliness [hazard ratio (HR) = 1.14, 95% CI = 1.11–1.17] was associated with an increased risk of overall MSK disorders, whereas social isolation was associated with a modestly lower risk of MSK disorders (HR = 0.96, 95% CI = 0.94–0.99). Loneliness ranked higher in relative strength than most of the traditional risk factors. Significant interactions (on both additive and multiplicative scales) between loneliness and traditional risk factor control were observed. When stratified by genetic risk, loneliness was consistently associated with an increased risk of overall MSK disorders across strata. Joint analysis indicated the absence of loneliness to offset the genetic risk of overall MSK disorders. When extending to five specific conditions, largely consistent patterns of results were observed.

**Conclusion:**

Loneliness is associated with increased MSK disorder risk, shows an interaction with the degree of risk factor control, and its absence may modify genetic predisposition. These findings support targeting loneliness to reduce the burden of MSK disorders.

Rheumatology key messages

- Loneliness, but not social isolation, increased the risk of musculoskeletal disorders.
- Loneliness ranked above most traditional risk factors for musculoskeletal disorders and interacted with them.
- The effects of loneliness on musculoskeletal disorder risk persisted across genetic risk strata, supporting the targeting of loneliness in risk reduction.

## Introduction

Musculoskeletal (MSK) disorders represent a major global health challenge, ranking as the leading cause of years lived with disability and the fifth-highest contributor to disability-adjusted life years worldwide [1]. The considerable burden of these disorders is largely attributed to five key conditions, including OA, gout, RA, low back pain, and neck pain [1–3]. Their profound economic and social costs underscore the critical need to identify modifiable determinants for developing effective, targeted prevention strategies.

Despite extensive research establishing traditional risk factors for MSK disorders—such as obesity [4–10], physical inactivity [4–6], poor sleep [11], unhealthy diet [4, 8–10], smoking [4–6, 8, 10], and alcohol consumption [4, 8–10]—a significant residual risk remains, even among individuals with optimal control of these metabolic and behavioral determinants, suggesting involvement of additional pathways. Psychosocial factors, particularly loneliness and social isolation, have emerged as compelling contributors. These constructs, despite sharing similarities to some extent, act differently—loneliness denotes the subjective, distressing perception of inadequate social connections, while social isolation refers to the objective absence of social relationships [12, 13]. Importantly, they may influence MSK health through distinct pathways: loneliness via neuroendocrine dysregulation [14], chronic low-grade inflammation [15], and altered pain perception [16], and social isolation primarily through behavioral mechanisms such as reduced physical activity [17, 18].

Although loneliness and social isolation are linked to various health outcomes [12, 19–22], their roles in MSK disorders remain poorly defined. Cross-sectional studies suggest loneliness is associated with higher odds of OA, RA, and low back pain, while evidence for social isolation is mixed, showing both negative and null associations across conditions [23–25]. Prospective evidence is scarce and contradictory; a single recent study identified social isolation, but not loneliness, as a risk factor for incident RA [26]. These discrepancies likely arise from methodological limitations, including cross-sectional designs, single-disease focus, and unmeasured confounding. Furthermore, the relative importance of these psychosocial factors compared with traditional determinants, and whether their effects are independent of or synergistic with traditional risk factors, remain unknown.

Genetic predisposition is a well-established determinant of MSK disorders, with heritability estimates ranging from 20% to 60% [6, 27–30]. While gene–environment interactions have been well documented for traditional lifestyle factors, such as obesity, sleep patterns, and physical activity [31, 32], it remains unclear whether psychosocial exposures, including loneliness and social isolation, interact with genetic predisposition in the development of MSK disorders. Elucidating these interactions could advance precision prevention by identifying high-risk subgroups with combined genetic and psychosocial vulnerability.

To address these gaps, we conducted a large-scale prospective cohort study using UK Biobank (UKB) data, aiming to (1) investigate associations of loneliness and social isolation with overall and condition-specific MSK disorders; (2) quantify their importance relative to traditional risk factors; and (3) assess their joint effects and interactions with traditional risk factor control and genetic susceptibility.

## Methods

### Study design and population

This study constitutes a secondary analysis of the UKB, a prospective cohort study that recruited ∼500 000 participants aged 37–73 years from 22 assessment centres across the UK between 2006 and 2010. Participants were identified through the National Health Service patient register and invited by mail if they lived within approximately 40 km of an assessment centre. At the initial visit, all participants completed a touchscreen questionnaire, underwent a physical examination, and provided biological samples. Details have been described elsewhere [33].

**Figure 1:**
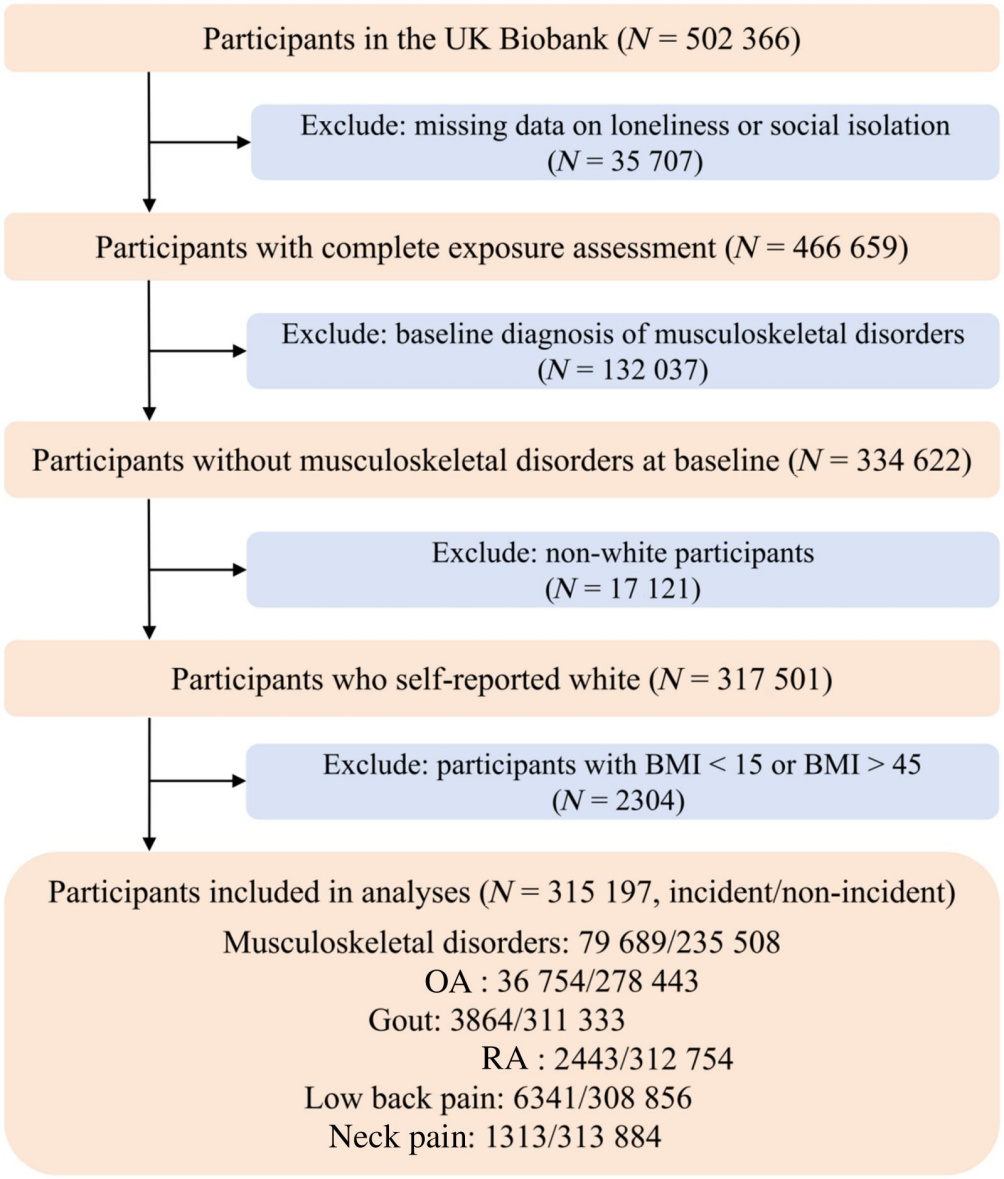
Flowchart of participant selection in the UK Biobank

In this study, we excluded those with missing data on loneliness or social isolation (*n* = 35 707), a prevalent diagnosis of MSK disorders at or prior to baseline (*n* = 132 037), non-white ethnicity (*n* = 17 121) [34], and extreme BMI (<15 or >45 kg/m^2^, *n* = 2304). This restriction to white participants was applied to align with the European-ancestry–derived polygenic risk score (PRS) and minimize population stratification bias. Finally, a total of 315 197 participants remained in the primary analysis (Fig. 1). For the analyses involving traditional risk factors, we further restricted the study sample to the 250 372 participants with complete data on all six risk factors, and for the genetic susceptibility analyses, we restricted the study sample to the 257 376 participants who passed genotyping quality control checks. The baseline characteristics of the included and excluded participants are presented in Supplementary Table S1–2.

### Assessment of loneliness and social isolation

Loneliness and social isolation were assessed using composite scales from baseline questionnaires, consistent with previously established methodologies in the UKB [12, 19–21, 26]. The loneliness scale was constructed from two questions, ‘Do you often feel lonely?’ and ‘How often are you able to confide in someone close to you?’ A high-risk response for loneliness was defined as answering ‘yes’ to often feeling lonely and confiding in someone less than once a month. Specifically, one point was assigned to each high-risk factor, resulting in a composite score ranging from 0 to 2, with higher scores indicating greater loneliness. The social isolation scale was constructed from three questions, ‘Including yourself, how many people are living together in your household?’, ‘How often do you visit friends or family or have them visit you?’ and ‘Which of the following (sports club or gym, pub or social club, religious group, adult education class, other group activity) do you engage in once a week or more often?’. A high-risk response for social isolation was defined for each item as: living alone, having friends or family visit less than once a month, and no participating in social activity at least once a week. A point was assigned for each high-risk factor, resulting in a composite score ranging from 0 to 3, with higher scores indicating greater social isolation. Participants were further categorized as experiencing ‘loneliness’ if they had a loneliness score of 2, and experiencing ‘no loneliness’ if their score was <2. Similarly, participants were categorized as ‘isolated’ if they had a social isolation score ≥2, and ‘not isolated’ if their score was <2 [19]. The specific field IDs used for these variables are presented in Supplementary Table S3.

### Definition of traditional risk factor control

Based on previously well-established studies and clinical guidelines [4–11], the status of six modifiable factors was chosen to determine traditional risk factor control: BMI, sleep duration, physical activity, dietary pattern, smoking, and alcohol consumption. For each factor, well-controlled status was defined according to specific criteria: non-obesity, normal sleep duration, moderate or vigorous physical activity, healthy diet, non-current smoking, and non-current alcohol drinking. Each factor was assigned a binary score, with 1 indicating well controlled and 0 indicating not well controlled. These scores were summed to create a composite score for risk factor control, ranging from 0 to 6, with higher scores indicating more favourable overall risk factor management. Participants were then categorized into two groups based on this score: less well controlled (scores 0–4), and well controlled (scores 5–6). Detailed definitions for the level of control of each risk factor are shown in Supplementary Table S4.

### Ascertainment of outcomes

The primary outcome of the present study was the incidence of any MSK disorders. The secondary outcomes included the incidence of five specific, high-burden MSK conditions, including OA, gout, RA, low back pain, and neck pain. Outcome ascertainment was primarily based on data from hospital admission records, using diagnostic codes from the International Classification of Diseases, Ninth Revision (ICD-9) and Tenth Revision (ICD-10) codes. Additional cases were identified through self-reported data. Detailed coding information [2] is shown in Supplementary Table S5. Furthermore, the index date (time zero) was defined as the date of the baseline assessment centre visit, at which all exposure and covariate data were collected. The follow-up time was calculated from the date of the baseline to the diagnosis of a MSK disorder, death, loss to follow-up, or end of follow-up (31 October 2022), whichever came first.

### Construction of genetic risk score

The detailed methods of genotyping, imputation, and quality control in UKB have been described elsewhere [35]. The PRS of RA was derived from the ‘Standard PRS’ provided by the UKB PRS Release [36]. The remaining PRSs were calculated using methods established previously [37]. Briefly, we selected independent single nucleotide polymorphisms (SNPs) associated with disease risk reported by published genome-wide association studies (GWASs) [38–40], weighted by their respective β coefficients and normalized accordingly (Supplementary Table S6). A metaPRS capturing overall MSK genetic risk was constructed by combining standardized trait-specific PRSs using elastic-net logistic regression with 10-fold cross-validation, adjusting for age, sex, genetic arrays, and the top 10 genetic principal components (PCs) [41] (details in Supplementary Data S1). The participants were further classified into low (quintile 1), intermediate (quintiles 2–4), and high (quintile 5) genetic risk groups.

### Assessment of covariates

Potential covariates were selected at baseline based on previous literature and expert knowledge [4, 10, 20, 42, 43]. Covariates included age, sex, assessment centre, education level, current employment status, Townsend deprivation index (TDI), grip strength, systolic blood pressure, diastolic blood pressure, antihypertensive medication use, antidiabetic medication use, statin use, smoking status, alcohol consumption status, physical activity, sleep duration, healthy diet score, BMI, the top 10 PCs, and genotype batch. Detailed information is provided in Supplementary Table S7.

### Statistical analysis

Baseline characteristics were summarized as mean ± s.d. or median (IQR) for continuous variables and as a percentage for categorical variables. Differences across exposure groups were assessed using one-way ANOVA for continuous variables and χ^2^ tests for categorical variables. Missing data were handled by median imputation for continuous variables and creation of separate categories for categorical variables.

In the survival analyses, Kaplan–Meier curves were applied to visualize the cumulative incidence of MSK disorders. Cox proportional hazards models were used to examine the association of loneliness and social isolation with risk of MSK disorders. Hazard ratios (HRs) with corresponding 95% CIs were evaluated through two models: model 1 was adjusted for age and sex; model 2 was a fully adjusted model that additionally included assessment centre, education level, current employment status, TDI, grip strength, systolic blood pressure, diastolic blood pressure, antihypertensive medication use, antidiabetic medication use, statin use, smoking status, alcohol consumption status, physical activity, sleep duration, healthy diet score and BMI. We further explored associations of individual scale components with MSK risk and assessed trends by modelling the scores as continuous variables. To estimate how important loneliness or isolation is in predicting MSK disorders, we compared their relative importance with traditional risk factors by calculating the *R*^2^ values [19, 44]. We also computed the explainable log-likelihood contribution for each risk factor to ensure consistency of results [19].

We further examined the joint effects of psychosocial and traditional risk factors by creating combined exposure categories and conducting stratified analyses across different risk strata. We assessed potential effect modification on two scales: multiplicative interactions (assessing whether joint effects differ from the product of individual effects) using likelihood ratio tests, and additive interactions (assessing whether combined effects exceed the sum of individual effects) using the relative excess risk due to interaction (RERI), attributable proportion (AP), and synergy index (S) with 95% CI, using the lowest-risk stratum as the reference [45]. For these analyses, models were not adjusted for the specific risk factors involved in the composite score to avoid over-adjustment. Similar joint, stratified, and interaction methodologies evaluated the interplay between psychosocial factors and PRS. All analyses involving genetic data were additionally adjusted for the top 10 PCs and genetic batch.

Several sensitivity analyses were conducted to assess the robustness of the results. First, we mutually adjusted for loneliness and social isolation to determine whether their associations were independent. Second, we excluded incident cases diagnosed within the first 2 years of follow-up to minimize reverse causation. Third, we restricted the analysis to participants with complete data on all covariates. Fourth, we further adjusted for occupational physical workload and subclinical musculoskeletal symptoms. Fifth, we conducted a sensitivity analysis excluding traditional risk factors to address potential over-adjustment.

All statistical analyses were conducted using R version 4.4.2 software. A two-sided *P-*value of <0.05 was considered significant.

## Results

### Population characteristics

The baseline characteristics of the study population are shown in Table 1 and Supplementary Table S8. A total of 315 197 participants were included in the primary analysis, with a mean (s.d.) age of 55.9 (8.1) years and 146 126 (46.4%) being males. Participants with a higher level of loneliness or social isolation were more likely to be male, have lower educational attainment, higher deprivation, lower grip strength, and greater use of antihypertensive, antidiabetic, and statin medications. They also tended to be current smokers, exercise less, have unhealthier sleep duration, and higher BMI.

**Table 1:** Baseline characteristics of participants in the UK Biobank by loneliness status.

Baseline characteristics	Total	No loneliness	Loneliness	*P*_value_
**No. of participants**	315 197	297 455	17 742	
**Age, years**	55.9 ± 8.1	55.9 ± 8.1	55.4 ± 7.9	<0.001
**Male, *n* (%)**	146 126 (46.4)	137 238 (46.1)	8888 (50.1)	<0.001
**Assessment centre**				0.022
England, *n* (%)	276 834 (87.8)	261 363 (87.9)	15 471 (87.2)	
Scotland, *n* (%)	24 342 (7.7)	22 919 (7.7)	1423 (8.0)	
Wales, *n* (%)	14 021 (4.4)	13 173 (4.4)	848 (4.8)	
**College/university degree, *n* (%)**	110 961 (35.2)	106 194 (35.7)	4767 (26.9)	<0.001
**Employed, *n* (%)**	196 820 (62.4)	186 107 (62.6)	10 713 (60.4)	<0.001
**TDI**	−1.6 ± 2.9	−1.6 ± 2.9	−0.8 ± 3.3	<0.001
**Grip strength, kg**	31.6 ± 10.7	31.6 ± 10.7	31.1 ± 10.9	<0.001
**SBP, mmHg**	137.4 ± 18.7	137.5 ± 18.7	136.4 ± 18.3	<0.001
**DBP, mmHg**	82.1 ± 10.2	82.1 ± 10.1	82.1 ± 10.4	0.888
**Antihypertensive medication use, *n* (%)**	56 681 (18.0)	53 042 (17.8)	3639 (20.5)	<0.001
**Antidiabetic medication use, *n* (%)**	2727 (0.9)	2452 (0.8)	275 (1.5)	<0.001
**Statin use, *n* (%)**	47 244 (15.0)	44 085 (14.8)	3159 (17.8)	<0.001
**Current smoker, *n* (%)**	31 604 (10.0)	28 623 (9.6)	2981 (16.8)	<0.001
**Current drinker, *n* (%)**	296 839 (94.2)	280 585 (94.3)	16 254 (91.6)	<0.001
**Physical activity, MET-min/week**	2637.0 ± 2609.9	2640.0 ± 2598.1	2585.0 ± 2808.2	0.017
**Sleep duration**				<0.001
Normal, 7–8 hours/day, *n* (%)	222 155 (70.5)	211 905 (71.2)	10 250 (57.8)	
Short, <7 hours/day, *n* (%)	69 870 (22.2)	63 882 (21.5)	5988 (33.8)	
Long, >8 hours/day, *n* (%)	22 190 (7.0)	20 821 (7.0)	1369 (7.7)	
**Healthy diet score**	3.0 (2.0, 4.0)	3.0 (2.0, 4.0)	3.0 (2.0, 4.0)	<0.001
**BMI**	27.0 ± 4.4	26.9 ± 4.3	27.7 ± 4.9	<0.001

Abbreviations: TDI: Townsend deprivation index; SBP: systolic blood pressure; DBP: diastolic blood pressure; MET: metabolic equivalent of task; healthy diet score: range 0–5 points; positively linked with the level of adherence to a healthy diet.

### Loneliness or social isolation with the risk of MSK disorders

During a median follow-up of 13.3 years [interquartile range (IQR) 11.5–14.2 years], 79 689 incident cases of MSK disorders were identified. Loneliness was associated with a significantly higher risk of incident overall MSK disorders, with HRs of 1.26 (95% CI = 1.22–1.29) after age and sex adjustment and 1.14 (95% CI = 1.11–1.17) after full multivariable adjustment (Table 2). This association followed a dose–gradient relationship (*P*_trend_ < 0.001) (Supplementary Table S9) and was consistent across all specific MSK conditions (Table 2), with the strongest risks observed for low back pain (HR = 1.32, 95% CI = 1.20–1.44) and neck pain (HR = 1.30, 95% CI = 1.06–1.58). Consistent patterns were obtained from Kaplan–Meier curves (Supplementary Fig. S1). Both components of loneliness—feeling lonely (HR = 1.14, 95% CI = 1.12–1.16) and being unwilling to confide in others (HR = 1.04, 95% CI = 1.03–1.06)—were independently associated with higher MSK risk (Supplementary Table S9). In contrast, social isolation showed a weak inverse association with overall MSK disorders (HR = 0.96, 95% CI = 0.94–0.99) and OA (HR = 0.89, 95% CI = 0.86–0.93) but was not associated with other specific MSK conditions (Supplementary Table S10).

**Table 2:** Associations of loneliness with subsequent risk for musculoskeletal disorders.

	*N*	Cases/Person-years	Model 1 HR (95% CI)	Model 2 HR (95% CI)
**Overall MSK**				
No loneliness	297 455	74 499/3 523 290.7	1.00 Ref.	1.00 Ref.
Loneliness	17 742	5190/203 250.6	**1.26 (1.22–1.29)**	**1.14 (1.11–1.17)**
**OA**				
No loneliness	297 455	34 434/3 782 887.7	1.00 Ref.	1.00 Ref.
Loneliness	17 742	2320/222 150.1	**1.21 (1.16–1.26)**	**1.09 (1.04–1.13)**
**Gout**				
No loneliness	297 455	3587/3 962 484.0	1.00 Ref.	1.00 Ref.
Loneliness	17 742	277/234 281.0	**1.37 (1.21–1.55)**	**1.13 (1.00–1.28)**
**RA**				
No loneliness	297 455	2262/3 966 137.8	1.00 Ref.	1.00 Ref.
Loneliness	17 742	181/234 437.9	**1.42 (1.22–1.65)**	**1.20 (1.03–1.39)**
**Low back pain**				
No loneliness	297 455	5811/3 947 422.4	1.00 Ref.	1.00 Ref.
Loneliness	17 742	530/232 594.7	**1.59 (1.46–1.74)**	**1.32 (1.20–1.44)**
**Neck pain**				
No loneliness	297 455	1203/3 970 414.9	1.00 Ref.	1.00 Ref.
Loneliness	17 742	110/234 692.6	**1.58 (1.30–1.92)**	**1.30 (1.06–1.58)**

Model 1: adjusted for age and sex. Model 2: additionally adjusted for assessment centre, education level, current employment status, Townsend deprivation index, grip strength, systolic blood pressure, diastolic blood pressure, antihypertensive medication use, antidiabetic medication use, statin use, smoking status, alcohol consumption status, physical activity, sleep duration, healthy diet score and BMI, based on model 1. Bold values indicate statistically significant associations (*P* < 0.05). Abbreviations: MSK: musculoskeletal disorders; HR: hazard ratio.

### The relative importance of loneliness compared with traditional risk factors in predicting MSK disorders

**Figure 2:**
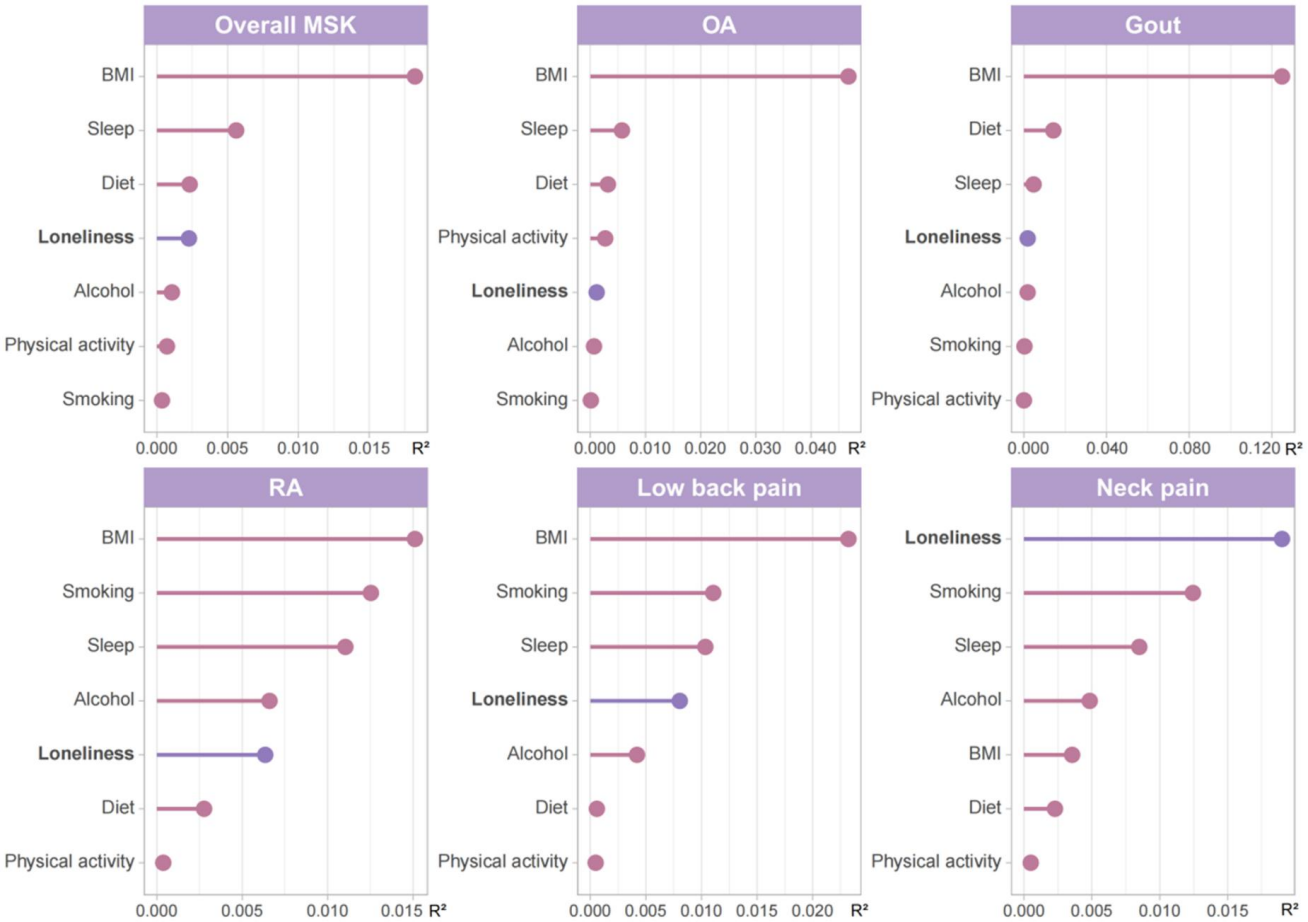
Relative importance of loneliness and traditional risk factors for predicting musculoskeletal disorders. Analysis was restricted to 250 372 participants who had complete data on loneliness and risk factor control. Abbreviations: MSK: musculoskeletal disorders

For overall MSK disorders, loneliness ranked as the fourth most influential risk factor overall—exceeded only by BMI and sleep duration, comparable with diet, but stronger than alcohol consumption, physical activity, and smoking (Fig. 2). This moderate-to-high predictive strength was consistently observed across all specific MSK conditions. The robustness of findings was further confirmed by explained log-likelihood analysis (Supplementary Table S11), which yielded risk factor rankings largely consistent with the variance explained (*R*^2^) approach.

### Joint association of loneliness and the degree of risk factor control with MSK disorders

Participants experiencing both loneliness and less-well-controlled traditional risk factors showed the highest risk of overall MSK disorders (HR = 1.31, 95% CI = 1.26–1.36) compared with those with no loneliness and well-controlled risk factors (Table 3). In the stratified analysis, among participants with well-controlled risk factors, the association between loneliness and overall MSK risk was not significant (HR = 1.07, 95% CI = 0.97–1.18). In contrast, among participants with less-well-controlled risk factors, loneliness was associated with a significant 20% increased risk (HR = 1.20, 95% CI = 1.16–1.24). This synergistic relationship was confirmed by a statistically significant interaction on both the additive (RERI = 0.15, 95% CI = 0.04–0.26; AP = 12%, 95% CI = 3%-20%; *P = *0.003) and multiplicative scales (*P = *0.019). Similar patterns of elevated risk were observed across all five specific MSK conditions. However, a significant additive interaction was identified only for low back pain (RERI = 0.57, 95% CI = 0.14–1.00; AP = 31%, 95% CI = 9%-53%; *P = *0.004).

**Table 3:** Joint and stratified analysis of loneliness and the degree of risk factor control with MSK.

		Overall MSK	OA	Gout	RA	Low back pain	Neck pain
		HR (95% CI)	HR (95% CI)	HR (95% CI)	HR (95% CI)	HR (95% CI)	HR (95% CI)
**Joint effects analysis**							
Risk factor control	Loneliness						
Well	No	1.00 Ref.	1.00 Ref.	1.00 Ref.	1.00 Ref.	1.00 Ref.	1.00 Ref.
Well	Yes	1.06 (0.97–1.17)	1.10 (0.96–1.27)	1.05 (0.62–1.80)	1.53 (0.95–2.46)	1.07 (0.75–1.53)	1.27 (0.62–2.59)
Less well	No	**1.09 (1.07–1.11)**	**1.17 (1.14–1.21)**	**1.43 (1.28–1.61)**	**1.18 (1.04–1.34)**	**1.22 (1.12–1.32)**	1.17 (0.98–1.39)
Less well	Yes	**1.31 (1.26–1.36)**	**1.34 (1.26–1.42)**	**1.64 (1.37–1.97)**	**1.38 (1.10–1.73)**	**1.86 (1.64–2.11)**	**1.86 (1.41–2.44)**
**Stratified analysis**							
Risk factor well controlled							
No loneliness		1.00 Ref.	1.00 Ref.	1.00 Ref.	1.00 Ref.	1.00 Ref.	1.00 Ref.
Loneliness		1.07 (0.97–1.18)	1.11 (0.97–1.28)	1.04 (0.61–1.77)	1.57 (0.97–2.53)	1.08 (0.75–1.55)	1.27 (0.62–2.59)
Risk factor less well controlled							
No loneliness		1.00 Ref.	1.00 Ref.	1.00 Ref.	1.00 Ref.	1.00 Ref.	1.00 Ref.
Loneliness		**1.20 (1.16–1.24)**	**1.14 (1.08–1.20)**	1.15 (0.99–1.33)	1.16 (0.94–1.42)	**1.52 (1.37–1.70)**	**1.58 (1.25–2.00)**
No loneliness							
Well controlled		1.00 Ref.	1.00 Ref.	1.00 Ref.	1.00 Ref.	1.00 Ref.	1.00 Ref.
Less well controlled		**1.09 (1.07–1.11)**	**1.17 (1.13–1.21)**	**1.43 (1.27–1.60)**	**1.18 (1.04–1.34)**	**1.22 (1.12–1.32)**	1.17 (0.98–1.39)
Loneliness							
Well controlled		1.00 Ref.	1.00 Ref.	1.00 Ref.	1.00 Ref.	1.00 Ref.	1.00 Ref.
Less well controlled		**1.22 (1.11–1.35)**	**1.22 (1.05–1.42)**	1.65 (0.95–2.85)	0.90 (0.54–1.50)	**1.71 (1.18–2.47)**	1.42 (0.68–2.97)
***P* for multiplicative interaction**		**0.019**	0.638	0.765	0.317	0.052	0.545
***RERI*** (95% CI)		**0.15 (0.04–0.26)**	0.06 (−0.11, 0.24)	0.16 (−0.45, 0.77)	−0.33 (−1.11, 0.44)	**0.57 (0.14, 1.00)**	0.42 (−0.56, 1.40)
***AP*** (95% CI)		**0.12 (0.03–0.20)**	0.05 (−0.08, 0.18)	0.09 (−0.27, 0.46)	−0.24 (−0.82, 0.33)	**0.31 (0.09, 0.53)**	0.23 (−0.29, 0.74)
***S*** (95% CI)		1.99 (1.00–3.97)	1.24 (0.67, 2.29)	1.32 (0.39, 4.50)	0.53 (0.16, 1.81)	2.99 (0.74, 12.12)	1.96 (0.23, 16.73)

Analysis was restricted to 250 372 participants who had complete data on loneliness and risk factor control. The six risk factor controls included non-obesity, normal sleep duration, moderate or vigorous physical activity, healthy diet, non-current smoking and non-current alcohol drinking (range from 0 to 6). The degree of traditional risk factor control was classified as less well controlled (0–4 risk factor control), and well controlled (5–6 risk factor control). Models were adjusted for age, sex, assessment centre, education level, current employment status, Townsend deprivation index, grip strength, systolic blood pressure, diastolic blood pressure, antihypertensive medication use, antidiabetic medication use and statin use. Bold values indicate statistically significant associations (*P* < 0.05). Abbreviations: MSK: musculoskeletal disorders; HR: hazard ratio; RERI: relative excess risk due to interaction; AP: attributable proportion due to interaction; S: the synergy index.

### Joint association of loneliness and genetic risk with MSK disorders

**Figure 3:**
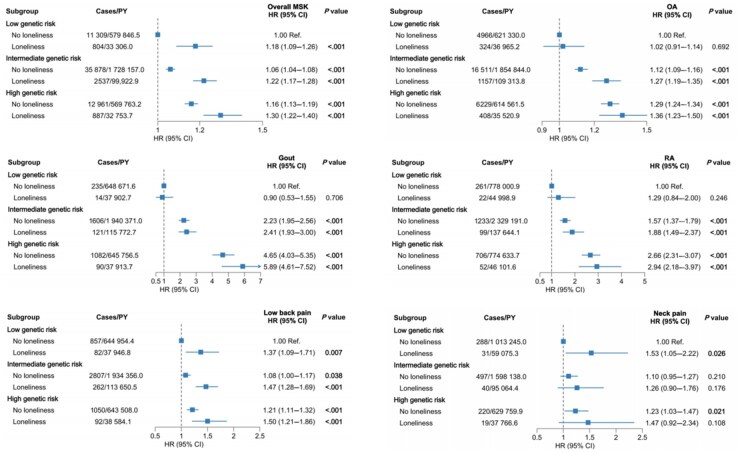
Joint association of loneliness and PRS with MSK risk. Analysis was restricted to 257 376 participants who passed genotyping quality control checks. The analysis was performed in Model 2 (adjusted with age, sex, assessment centre, education level, current employment status, Townsend deprivation index, grip strength, systolic blood pressure, diastolic blood pressure, antihypertensive medication use, antidiabetic medication use, statin use, smoking status, alcohol consumption status, physical activity, sleep duration, healthy diet score and BMI), with additional adjustment for genetic principal components (PCs) 1–10, genotyping batch. Abbreviations: MSK: musculoskeletal disorders; PRS: polygenic risk score; UKB: UK biobank; PY: person years; HR: hazard ratio

As shown in Fig. 3, participants with both high metaPRS and loneliness exhibited the highest risk of overall MSK disorders (HR = 1.30, 95% CI = 1.22–1.40) compared with those with low metaPRS and no loneliness. Notably, participants without loneliness but with intermediate (HR = 1.06, 95% CI = 1.04–1.08) or high metaPRS (HR = 1.16, 95% CI = 1.13–1.19) showed a risk level that was similar or even lower than those with loneliness but low metaPRS (HR = 1.18, 95% CI = 1.09–1.26). A similar pattern was observed for low back pain, but less evident for other specific MSK conditions.

In stratified analyses, loneliness was consistently associated with an increased risk of overall MSK disorders across all genetic risk strata (Supplementary Table S12). Similar patterns were observed for individual MSK conditions, although not all reached statistical significance. Neither multiplicative nor additive interaction was observed (Supplementary Tables S12–13).

### Sensitivity analyses

Results remained consistent across all sensitivity analyses: mutually adjusting for loneliness and isolation (Supplementary Table S14), excluding incident MSK disorders occurring within the first 2 years of follow-up (Supplementary Table S15), restricting the analysis to participants with complete data on all covariates (Supplementary Table S16), additional adjusting for occupational physical workload (Supplementary Table S17) and subclinical musculoskeletal symptoms (Supplementary Table S18), and excluding traditional risk factors (Supplementary Table S19).

## Discussion

In this large prospective cohort study, loneliness, but not social isolation, was significantly associated with increased risk of incident MSK disorders, with a predictive strength surpassing that of several traditional risk factors. Loneliness acted synergistically with poor traditional risk factor control on both additive and multiplicative scales, and its absence appeared to mitigate genetic predisposition to MSK disorders. Associations were broadly consistent across the five specific MSK conditions examined.

Our findings establish loneliness as a robust, independent risk factor for a broad spectrum of MSK disorders. This conclusion is supported by a limited but convergent body of prior research, including cross-sectional studies reporting associations between loneliness and higher odds of OA and RA across age groups [23, 24], as well as studies linking loneliness to low back pain and neck pain [25, 46–48]. While a recent UK Biobank study found a detrimental, albeit non-significant, trend towards association between loneliness and RA, methodological differences likely account for this discrepancy [26]. Crucially, our study extends prior condition-specific research by demonstrating loneliness as a systemic determinant of MSK health, suggesting a shared upstream pathway and justifying the use of a composite outcome. Furthermore, the particularly strong associations observed for low back pain and neck pain may be attributed to the sensitivity of these regions to postural alterations and muscle tension, both of which can be exacerbated by the physiological stress response associated with loneliness [14–16]. Thus, addressing loneliness represents a novel preventive strategy for MSK.

For the first time, loneliness was positioned as a risk factor for MSK disorders with predictive importance comparable with or higher than that of several established traditional risk factors, underscoring its clinical significance. We further identified a synergistic interaction between loneliness and poor traditional risk factor control, with ∼12% of MSK cases in this high-risk group attributable to their interaction—an effect particularly salient for low back pain. Collectively, our findings advocate for a holistic prevention strategy integrating psychological well-being and lifestyle management. As loneliness denotes subjective perception rather than objective social isolation, effective mitigation strategies should prioritize enhancing perceived social connectedness through cognitive–behavioural interventions, social prescribing, and therapies addressing maladaptive social cognition [49].

Although no significant interactions were identified between loneliness and genetic risk, a key finding emerged: for overall MSK disorders and low back pain, individuals with high genetic susceptibility but no loneliness exhibited lower risk than those with low genetic risk but high loneliness, suggesting that loneliness may counteract the detrimental effects of genetic susceptibility. This has implications for precision prevention: genetic profiling can identify individuals with elevated baseline risk, while loneliness reduction offers a universally applicable mitigation strategy across all genetic strata.

In contrast to loneliness, social isolation showed a weak inverse association with overall MSK risk, driven primarily by a reduced OA risk with no significant associations for other conditions. This divergence underscores their etiological distinction: loneliness, as a subjective psychosocial stressor, likely exerts direct biological harm through chronic inflammatory and neuroendocrine activation [14, 15], whereas social isolation may correlate with protective behavioural adaptations. For instance, reduced high-impact activity among socially isolated older adults may decrease cumulative joint stress and mechanical injury [10, 17]. The relationship between social isolation and MSK health thus appears highly condition-specific and warrants further investigation.

Despite the novel strengths of this large-scale prospective study regarding loneliness and MSK, several limitations warrant acknowledgment. First, residual confounding cannot be completely ruled out, and causal inferences cannot be established in an observational study. Second, the brief scales used to measure loneliness and social isolation may oversimplify these constructs; notably, social isolation did not capture structured social interactions such as those occurring in work or educational settings. Third, case ascertainment through inpatient records potentially missed milder cases managed in outpatient or primary care settings, particularly for pain-related conditions. Fourth, reverse causation cannot be fully excluded, as subclinical MSK symptoms at baseline may have influenced social engagement and perceived loneliness. Fifth, psychosocial exposures were assessed only at baseline, although supplementary analyses confirmed temporal stability in this subset (Supplementary Tables S20–21). Sixth, healthy volunteer bias may lead to underestimation of the observed associations, and the restriction to white participants limits not only the generalizability of our findings, but also the adequate representation of the increasingly ethnically diverse UK population. Finally, loneliness is a culturally specific phenomenon, and our interpretation may not have fully accounted for these cultural nuances. Future research would benefit from cross-cultural comparative studies across diverse sociocultural settings, qualitative approaches to better understand culturally specific experiences of loneliness, and closer collaboration with researchers familiar with the relevant sociocultural context to ensure more contextually grounded interpretations.

## Conclusion

In conclusion, loneliness was independently associated with increased MSK disorder incidence, with predictive strength comparable with that of traditional risk factors, and there were consistent associations across genetic susceptibility strata. A synergistic interaction between loneliness and poor traditional risk factor control indicates that their combined presence creates disproportionate disease risk. These findings underscore the importance of integrating loneliness reduction into conventional MSK prevention frameworks.

## Supplementary Material

keag326_Supplementary_Data

## Data Availability

The genetic and phenotypic UK Biobank data are available on application to the UK Biobank to any researcher worldwide (www.ukbiobank.ac.uk).
