# Supplementary material for: Loneliness, traditional risk factor control, genetic predisposition, and development of musculoskeletal disorders
Source: Rheumatology (Oxford). 2026 Jun 26;65(7):keag326. doi: 10.1093/rheumatology/keag326 (PMC13394693; doi:10.1093/rheumatology/keag326)
Supplement: keag326_Supplementary_Data [file keag326_supplementary_data.docx]

**Loneliness, traditional risk factor control, genetic predisposition, and development of musculoskeletal disorders**

**Table of contents**

**Supplementary Data S1** The construction of genetic risk score.

**Table S1.** Comparison of baseline characteristics between participants included and excluded from the traditional risk factor analyses in the UK Biobank.

**Table S2.** Comparison of baseline characteristics between participants included and excluded from the genetic analyses in the UK Biobank.

**Table S3.** Assessment of loneliness and social isolation in the UK Biobank.

**Table S4.** Definitions of traditional risk factor control for musculoskeletal disorders.

**Table S5.** Specific diagnostic criteria for diseases in UK Biobank.

**Table S6.** Information of SNPs associated with the risk of OA, gout, low back pain and neck pain reported by previous genome-wide association studies.

**Table S7.** Detailed information on covariates.

**Table S8.** Baseline characteristics of participants in the UK Biobank by social isolation status.

**Table S9.** Associations of the scores and items of loneliness with long-term risk of musculoskeletal disorders.

**Table S10.** Associations of social isolation with subsequent risk for musculoskeletal disorders.

**Table S11.** Relative importance of loneliness and traditional risk factors for predicting musculoskeletal disorders, by estimation of explained log-likelihood explained by each predictor.

**Table S12.** Joint association and stratified analysis of loneliness and the degree of PRS on the risk of musculoskeletal disorders.

**Table S13.** Interaction effects between loneliness and PRS on the risk of musculoskeletal disorders in the UKB cohort.

**Table S14.** Multivariable-adjusted HRs (95% CI) of loneliness or social isolation for musculoskeletal disorders, after mutual adjustment in models included all covariates.

**Table S15.** Associations of loneliness and social isolation with subsequent risk for musculoskeletal disorders after excluding those occurring musculoskeletal disorders within the first two years in the UKB cohort (n = 304,834).

**Table S16.** Associations of loneliness and social isolation with subsequent risk for musculoskeletal disorders after excluding participants with missing covariates in the UKB cohort (n = 250,372).

**Table S17.** Associations of loneliness and social isolation with risk for musculoskeletal disorders with additional adjustment for occupational physical workload.

**Table S18.** Associations of loneliness and social isolation with risk for musculoskeletal disorders with additional adjustment for subclinical musculoskeletal symptoms.

**Table S19**. Associations of loneliness and social isolation with subsequent risk for musculoskeletal disorders after excluding traditional risk factors.

**Table S20.** Stability of social isolation and loneliness across instances subset with repeated assessments.

**Table S21.** Proportion and trend of social isolation and loneliness by instance.

**Figure S1.** KM plots for the cumulative risks of musculoskeletal disorders between groups of no-loneliness and loneliness in the UKB cohort.

**Supplementary Data S1** The construction of genetic risk score.

The polygenic risk score (PRS) of RA was derived from “Standard PRS” provided by the UKB PRS Release, which has been validated with satisfactory predictive accuracy. The remaining PRSs were calculated using methods established previously. Briefly, we selected independent single nucleotide polymorphisms (SNPs) associated with disease risk reported by published GWASs: a total of 100 SNPs for OA, 291 SNPs for gout, 14 SNPs for low back pain, and 2 SNPs for neck pain. The information of SNPs is provided in Table S6. The PRSs were derived by weighting the number of effect alleles at each SNP locus (0, 1, or 2) using their respective β coefficients derived from published GWASs, summing these weighted values and normalizing by sum of β coefficients. A higher PRS indicated a higher genetic predisposition to the respective disorder.

A metaPRS was constructed to evaluate the genetic risk of overall MSK disorders by combining trait-specific PRSs. Each PRS was standardized to zero mean and unit SD. We then split the UKB into a training set and a testing set. Using the training set, we employed elastic-net logistic regression with 10-fold cross-validation via the R package “glmnet” to model the association between the trait-specific PRSs and overall MSK disorders, adjusting for age, sex, genetic arrays, and the top 10 genetic principal components (PCs). Multiple penalty models were evaluated, with the highest area under the receiver operating characteristic curve (AUC) model being selected and validated on the testing set. The metaPRS was calculated using a weighted average of the standardized trait-specific PRSs:

PRS_meta_ =
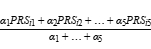
.

where
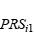
,…, and
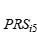
 are the five zero mean and unit variance standardized PRSs for the *i* th individual;
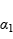
,…, and
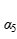
 are the coefficients for each of the five PRSs.

Participants were further classified into low (quintile 1), intermediate (quintiles 2-4), and high (quintile 5) genetic risk groups.

| **Table S1. Comparison of baseline characteristics between participants included and excluded from the traditional risk factor analyses in the UK Biobank.** | | | |
| --- | --- | --- | --- |
| **Baseline characteristics** | **Total**  **(N = 315,197)** | **Complete**  **(N = 250,372)** | **Incomplete**  **(N = 64,825)** |
| **Age, years** | 56.5 ± 8.1 | 55.6 ± 8.1 | 57.0 ± 8.0 |
| **Male, n (%)** | 229,068 (45.6) | 121,149 (48.4) | 24,977 (38.5) |
| **Assessment center** |  |  |  |
| England, n (%) | 445,725 (88.7) | 220,109 (87.9) | 56,725 (87.5) |
| Scotland, n (%) | 35,837 (7.1) | 19,303 (7.7) | 5,039 (7.8) |
| Wales, n (%) | 20,804 (4.1) | 10,960 (4.4) | 3,061 (4.7) |
| **Current smoker, n (%)** | 52,960 (10.5) | 23,978 (9.6) | 7,626 (11.9) |
| **Current drinker, n (%)** | 460,241 (91.6) | 236,937 (94.6) | 59,902 (92.6) |
| **Physical activity, METs** | 10.8 ± 4.2 | 10.8 ± 4.7 | 11.2 ± 5.1 |
| **Sleep duration** |  |  |  |
| Normal, 7-8 h/d, n (%) | 336,597 (67.0) | 179,064 (71.5) | 43,091 (67.5) |
| Short, <7 h/d, n (%) | 123,214 (24.5) | 54,284 (21.7) | 15,586 (24.4) |
| Long, >8 h/d, n (%) | 38,339 (7.6) | 17,024 (6.8) | 5,166 (8.1) |
| **Healthy diet score** | 3.0 (2.0, 4.0) | 3.0 (2.0, 4.0) | 3.0 (2.0, 4.0) |
| **BMI** | 27.4 ± 4.8 | 26.8 ± 4.3 | 27.3 ± 4.6 |
| **Grip strength, kg** | 30.6 ± 11.0 | 32.1 ± 10.7 | 29.4 ± 10.4 |
| **SBP, mmHg** | 138.0 ± 18.6 | 137.0 ± 18.5 | 139.1 ± 18.9 |
| **DBP, mmHg** | 82.3 ± 10.1 | 82.0 ± 10.2 | 82.4 ± 10.2 |
| **College/university degree, n (%)** | 161,102 (32.1) | 95,956 (38.3) | 15,005 (23.8) |
| **Employed, n (%)** | 287,048 (57.1) | 160,182 (64.0) | 36,638 (57.0) |
| **TDI** | -1.3 ± 3.1 | -1.7 ± 2.9 | -1.3 ± 3.1 |
| **Antihypertensive medication use, n (%)** | 103,982 (20.7) | 43,431 (17.3) | 13,250 (21.0) |
| **Antidiabetic medication use, n (%)** | 5,611 (1.1) | 2,087 (0.8) | 640 (1.0) |
| **Statin use, n (%)** | 86,874 (17.3) | 36,495 (14.6) | 10,749 (17.0) |
| Abbreviations: MET, metabolic equivalent of task; BMI, body mass index; SBP, systolic blood pressure; DBP, diastolic blood pressure; TDI, Townsend deprivation index. Healthy diet score: Range 0-5 points; positively linked with the level of adherence to a healthy diet. | | | |

| **Table S2. Comparison of baseline characteristics between participants included and excluded from the genetic analyses in the UK Biobank.** | | | |
| --- | --- | --- | --- |
| **Baseline characteristics** | **Total**  **(N = 315,197)** | **Complete**  **(N = 257,376)** | **Incomplete**  **(N = 57,821)** |
| **Age, years** | 56.5 ± 8.1 | 55.9 ± 8.1 | 56.1 ± 8.1 |
| **Male, n (%)** | 229,068 (45.6) | 120,537 (46.8) | 25,589 (44.3) |
| **Assessment center** |  |  |  |
| England, n (%) | 445,725 (88.7) | 226,022 (87.8) | 50,812 (87.9) |
| Scotland, n (%) | 35,837 (7.1) | 19,954 (7.8) | 4,388 (7.6) |
| Wales, n (%) | 20,804 (4.1) | 11,400 (4.4) | 2,621 (4.5) |
| **Current smoker, n (%)** | 52,960 (10.5) | 25,680 (10.0) | 5,924 (10.2) |
| **Current drinker, n (%)** | 460,241 (91.6) | 242,514 (94.2) | 54,325 (94.0) |
| **Physical activity, METs** | 10.8 ± 4.2 | 10.8 ± 4.3 | 10.8 ± 4.3 |
| **Sleep duration** |  |  |  |
| Normal, 7-8 h/d, n (%) | 336,597 (67.0) | 181,900 (70.7) | 40,255 (69.6) |
| Short, <7 h/d, n (%) | 123,214 (24.5) | 56,886 (22.1) | 12,984 (22.5) |
| Long, >8 h/d, n (%) | 38,339 (7.6) | 17,812 (6.9) | 4,378 (7.6) |
| **Healthy diet score** | 3.0 (2.0, 4.0) | 3.0 (2.0, 4.0) | 3.0 (2.0, 4.0) |
| **BMI** | 27.4 ± 4.8 | 26.9 ± 4.4 | 27.1 ± 4.4 |
| **Grip strength, kg** | 30.6 ± 11.0 | 31.7 ± 10.7 | 31.1 ± 10.7 |
| **SBP, mmHg** | 138.0 ± 18.6 | 137.4 ± 18.6 | 137.7 ± 18.7 |
| **DBP, mmHg** | 82.3 ± 10.1 | 82.1 ± 10.2 | 82.2 ± 10.1 |
| **College/university degree, n (%)** | 161,102 (32.1) | 93,088 (36.2) | 17,873 (30.9) |
| **Employed, n (%)** | 287,048 (57.1) | 161,163 (62.6) | 35,657 (61.7) |
| **TDI** | -1.3 ± 3.1 | -1.6 ± 2.9 | -1.6 ± 2.9 |
| **Antihypertensive medication use, n (%)** | 103,982 (20.7) | 45,996 (17.9) | 10,685 (18.5) |
| **Antidiabetic medication use, n (%)** | 5,611 (1.1) | 2,212 (0.9) | 515 (0.9) |
| **Statin use, n (%)** | 86,874 (17.3) | 38,341 (14.9) | 8,903 (15.4) |
| Abbreviations: MET, metabolic equivalent of task; BMI, body mass index; SBP, systolic blood pressure; DBP, diastolic blood pressure; TDI, Townsend deprivation index. Healthy diet score: Range 0-5 points; positively linked with the level of adherence to a healthy diet. | | | |

| **Table S3. Assessment of loneliness and social isolation in the UK Biobank.** | | | | |
| --- | --- | --- | --- | --- |
| **Exposures** | **Field ID** | **ACE touchscreen questions** | **Index** | **Responses** |
| Loneliness | 2020 | Do you often feel lonely? | 0 | No |
|  |  |  | 1 | Yes |
|  | 2110 | How often are you able to confide in someone close to you? | 0 | Almost daily |
|  |  |  |  | 2-4 times a week |
|  |  |  |  | About once a week |
|  |  |  |  | About once a month |
|  |  |  | 1 | Once every few months |
|  |  |  |  | Never or almost never |
| Social isolation | 709 | Including yourself, how many people are living together in your household? | 0 | Not living alone |
|  |  |  | 1 | Living alone |
|  | 1031 | How often do you visit friends or family or have them visit you? | 0 | Almost daily |
|  |  |  |  | 2-4 times a week |
|  |  |  |  | About once a week |
|  |  |  |  | About once a month |
|  |  |  | 1 | Once every few months |
|  |  |  |  | Never or almost never |
|  |  |  |  | No friends/family outside household |
|  | 6160 | Which of the following (sports club or gym, pub or social club, religious group, adult education class, other group activity) do you engage in once a week or more often? | 0 | Sports club or gym |
|  |  |  |  | Pub or social club |
|  |  |  |  | Religious group |
|  |  |  |  | Adult education class |
|  |  |  |  | Other group activity |
|  |  |  | 1 | None of the above |

| **Table S4. Definitions of traditional risk factor control for musculoskeletal disorders.** | | | | |
| --- | --- | --- | --- | --- |
| **Risk factor** | **Field ID** | **Descriptions** | **Control** | **Not control** |
| **BMI** | 21001 | Height and weight were measured during the assessment visit, and BMI was calculated as weight in kilograms divided by height in meters squared (kg/m^2^). | non-obesity  (BMI < 30 kg/m^2^) | obesity  (BMI ≥ 30 kg/m^2^) |
| **Sleep duration** | 1160 | Short: ≤ 6 hours, day; normal: 7 to 8 hours, day; and long: ≥ 9 hours, day | normal sleep duration  (7 to 8 hours per night) | abnormal sleep duration  (≤ 6 or ≥ 9 hours per night) |
| **Physical activity** | 22040 | Metabolic equivalent task—sum of minutes performing walking, moderate, and vigorous activity. We categorised participants by three mutually exclusive groups: low (< 600 metabolic equivalent (MET)-min/week), moderate (600 to < 3000 MET-min/week), and vigorous (≥ 3000 MET-min/week) PA based on a standard scoring criteria; the threshold at 600 MET-min/week is equivalent to reaching the recommended guidelines (150 min per week) for moderate-intensity PA. | moderate or vigorous physical activity | low physical activity |
| **Dietary patterns** | 1289/1299/1309/1319/1329/1339/1349/1369/1379/1389 | Calculated by using the following dietary factors: vegetable intake at least four tablespoons each day (median); fruits intake at least three pieces each day (median); fish intake at least twice each week (median); unprocessed red meat intake no more than twice each week (median); and processed meat intake no more than two each week (median). Each one point was given for each favorable diet factor, with the total diet score ranging from 0 to 5. Healthy diet pattern was defined as healthy diet score ≥ 4. | healthy diet, score ≥ 4 | unhealthy diet, score < 4 |
| **Smoking** | 20116 | never, previous, current | non-current smoking | current smoking |
| **Alcohol consumption** | 20117 | never, previous, current | non-current alcohol drinking | current alcohol drinking |

| **Table S5. Specific diagnostic criteria for diseases in UK Biobank.** | | | |
| --- | --- | --- | --- |
| **Diseases** | **Data-fields** | | |
|  | **ICD-10** | **ICD-9** | **Self-reported** |
| Overall MSK disorders | L93, M00–M02, M05–M06.9, M08.0–M08.8, M08, M10, M11–M13, M16, M17, M18, M19, M20–M25, M30–M35, M40–M43, M45–M46, M54.2, M54.3, M54.4, M54.5, M60–M63, M65–M68, M70–M73, M75–M79, M80–M85, M86, M87–M90, M91–M94, M95–M99 | 274, 710.0, 711, 712–713, 714–714.3, 714.8–714.9, 715, 716–719, 710.1–710.9, 737, 720–721, 723.1, 724, 725, 726–728, 729, 733.0–2, 730.1–730.3, 730.7–9, 731, 733.3–9, 732, 734–736, 738–739 | 1293–1295, 1297, 1308–1313, 1322, 1406–1407, 1464–1467, 1476–1478, 1532–1538, 1540–1542, 1544–1545, 1617–1624 |
| Osteoarthritis | M16, M17, M18, M19 | 715 | 1465 |
| Gout | M10 | 274 | 1466 |
| Rheumatoid arthritis | M05–M06.9, M08.0–M08.8 | 714–714.3, 714.8–714.9 | 1464 |
| Low back pain | M54.3, M54.4, M54.5 | 724 | 1534 |
| Neck pain | M54.2 | 723.1 | 1478 |

| **Table S6. Information of SNPs associated with the risk of OA, gout, low back pain and neck pain reported by previous genome-wide association studies.** | | | |
| --- | --- | --- | --- |
| **SNP** | **Effect allele** | **EAF** | **β** |
| **OA** |  |  |  |
| rs11588154 | T | 0.17 | -0.1864 |
| rs4411121 | T | 0.31 | 0.0677 |
| rs1327123 | C | 0.35 | -0.0944 |
| rs11588850 | A | 0.82 | -0.1393 |
| rs74676797 | A | 0.82 | 0.0488 |
| rs66989638 | A | 0.13 | 0.1133 |
| rs2276749 | T | 0.05 | -0.1508 |
| rs62242105 | A | 0.33 | -0.0305 |
| rs781661531 | T | 0.9997 | -2.2073 |
| rs747952496 | A | 0.0004 | 1.9488 |
| rs9835230 | A | 0.24 | 0.0677 |
| rs201194999 | T | 0.3 | -0.1278 |
| rs11729628 | T | 0.24 | -0.0305 |
| rs75686861 | A | 0.09 | 0.1133 |
| rs2066928 | A | 0.48 | -0.0408 |
| rs56132153 | A | 0.61 | 0.0677 |
| rs1560080 | A | 0.83 | -0.0943 |
| rs17615906 | T | 0.84 | -0.0513 |
| rs10062749 | T | 0.27 | 0.0770 |
| rs9396861 | A | 0.61 | 0.1222 |
| rs2038740 | T | 0.72 | -0.0619 |
| rs116934101 | A | 0.27 | 0.0583 |
| rs12667224 | A | 0.52 | -0.0305 |
| rs571734653 | A | 0.0003 | 1.7967 |
| rs7787744 | A | 0.67 | 0.0770 |
| rs76340814 | A | 0.05 | -0.1165 |
| rs79895530 | T | 0.13 | -0.1278 |
| rs7862601 | A | 0.62 | -0.0619 |
| rs10983775 | T | 0.54 | -0.0513 |
| rs10465114 | A | 0.22 | 0.0583 |
| rs3740129 | A | 0.46 | 0.0770 |
| rs10824456 | C | 0.58 | -0.0513 |
| rs3993110 | A | 0.61 | 0.0862 |
| rs1631174 | A | 0.34 | 0.0392 |
| rs72979233 | A | 0.75 | -0.0834 |
| rs10831475 | A | 0.81 | 0.0770 |
| rs10842226 | A | 0.42 | 0.0392 |
| rs7967762 | T | 0.16 | 0.1044 |
| rs1426371 | A | 0.27 | -0.0513 |
| rs58973023 | A | 0.49 | 0.0583 |
| rs28929474 | T | 0.02 | -0.2107 |
| rs746239049 | D | 0.21 | -0.1054 |
| rs12914479 | C | 0.66 | 0.0392 |
| rs6500609 | C | 0.11 | -0.0619 |
| rs227732 | T | 0.3 | 0.0583 |
| rs9908159 | T | 0.51 | 0.0392 |
| rs1039257158 | T | 0.0006 | 1.2865 |
| rs551471509 | T | 0.9996 | -1.7148 |
| rs8112559 | C | 0.89 | 0.1222 |
| rs9981884 | A | 0.49 | -0.0513 |
| rs11705555 | A | 0.76 | 0.0488 |
| rs12160491 | A | 0.71 | -0.0726 |
| rs11164653 | T | 0.41 | -0.0834 |
| 1:150214028 | D | 0.38 | 0.0392 |
| rs10797923 | T | 0.69 | 0.0488 |
| rs2605100 | A | 0.32 | 0.0677 |
| rs7581446 | T | 0.48 | -0.0513 |
| rs3771501 | A | 0.47 | 0.0392 |
| rs62182810 | A | 0.54 | 0.0296 |
| rs3774354 | A | 0.37 | 0.0953 |
| rs1530586 | T | 0.8 | 0.0862 |
| rs1913707 | A | 0.6 | 0.0862 |
| rs13107325 | T | 0.07 | 0.0770 |
| rs3884606 | A | 0.52 | -0.0408 |
| rs79220007 | T | 0.93 | -0.1054 |
| rs2856821 | T | 0.79 | 0.0488 |
| rs17288390 | T | 0.65 | -0.0834 |
| rs9475400 | T | 0.1 | 0.1398 |
| rs12209223 | A | 0.11 | 0.1989 |
| rs111844273 | A | 0.02 | 0.2311 |
| rs143083812 | T | 0.0011 | 1.1939 |
| rs11984666 | A | 0.2 | -0.1054 |
| rs10974438 | A | 0.65 | 0.0392 |
| rs72760655 | A | 0.33 | 0.0488 |
| rs1330349 | C | 0.59 | 0.0953 |
| rs1321917 | C | 0.41 | 0.0953 |
| rs62578126 | T | 0.37 | -0.0834 |
| rs1517572 | A | 0.41 | 0.0392 |
| rs67924081 | A | 0.74 | 0.0953 |
| rs34560402 | T | 0.06 | -0.1508 |
| rs1149620 | A | 0.44 | -0.0408 |
| rs7294636 | A | 0.37 | 0.1484 |
| rs10843013 | A | 0.78 | -0.1508 |
| rs17120227 | T | 0.07 | 0.1570 |
| rs7953280 | C | 0.5 | 0.0392 |
| rs753350451 | D | 0.2 | -0.0726 |
| rs1809889 | T | 0.28 | 0.0677 |
| rs4380013 | A | 0.19 | 0.0583 |
| rs11071366 | A | 0.61 | -0.1054 |
| rs12908498 | C | 0.54 | 0.0770 |
| rs9940278 | T | 0.43 | 0.0583 |
| rs34195470 | A | 0.45 | -0.0513 |
| rs216175 | A | 0.83 | 0.0392 |
| rs7212908 | A | 0.8 | -0.0943 |
| rs2716212 | A | 0.62 | -0.0726 |
| rs10405617 | A | 0.32 | 0.0296 |
| rs75621460 | A | 0.03 | 0.1906 |
| rs4252548 | T | 0.02 | 0.3293 |
| rs143384 | A | 0.59 | 0.0677 |
| rs9981408 | T | 0.23 | 0.0954 |
| **Gout** |  |  |  |
| rs2503700 | A | 0.6783 | 0.0374 |
| rs10754894 | A | 0.3037 | -0.0542 |
| rs565410312 | T | 0.8234 | -0.0381 |
| rs75511481 | T | 0.0517 | 0.0687 |
| rs79598313 | T | 0.0223 | 0.2492 |
| rs11587740 | C | 0.8404 | 0.0412 |
| rs6658904 | T | 0.443 | -0.0339 |
| rs469882 | A | 0.8071 | 0.0366 |
| rs7417952 | C | 0.3612 | -0.0350 |
| rs116831366 | A | 0.9883 | -0.1415 |
| rs2938616 | T | 0.67 | 0.0306 |
| rs12064784 | A | 0.022 | 0.1100 |
| rs9441166 | T | 0.4757 | 0.1004 |
| rs35950207 | T | 0.3028 | -0.0362 |
| rs9426886 | A | 0.5758 | 0.0887 |
| rs10799848 | A | 0.2335 | 0.0350 |
| rs1289004 | T | 0.4911 | 0.0333 |
| rs7552526 | T | 0.2412 | -0.0356 |
| rs633715 | T | 0.8037 | -0.0386 |
| rs142752230 | T | 0.2661 | 0.0388 |
| rs12727980 | T | 0.578 | -0.0278 |
| rs2808387 | C | 0.9648 | -0.0836 |
| rs61830291 | A | 0.9066 | 0.0555 |
| rs62106258 | T | 0.9522 | 0.1167 |
| rs12714415 | T | 0.8387 | 0.0800 |
| rs10165255 | A | 0.4316 | -0.0303 |
| rs807624 | T | 0.3459 | -0.033 |
| rs13406083 | A | 0.6457 | -0.0425 |
| rs188411445 | T | 0.0339 | 0.0884 |
| rs1260326 | T | 0.4159 | 0.1357 |
| rs7594951 | T | 0.1082 | -0.0461 |
| rs71446062 | A | 0.5906 | -0.0348 |
| rs77795433 | A | 0.0561 | -0.0734 |
| rs9798497 | T | 0.2883 | 0.0337 |
| rs1177293 | T | 0.4394 | 0.0348 |
| rs268124 | T | 0.7338 | -0.0308 |
| rs11693363 | A | 0.8813 | 0.0769 |
| rs764977 | T | 0.6621 | -0.0287 |
| rs17767183 | T | 0.3612 | -0.0474 |
| rs9973741 | A | 0.6112 | -0.0486 |
| rs17050272 | A | 0.42 | 0.0424 |
| rs34402378 | T | 0.5245 | 0.0378 |
| rs12989087 | C | 0.5161 | 0.0315 |
| rs1128249 | T | 0.3932 | -0.0360 |
| rs4140872 | T | 0.7582 | -0.0785 |
| rs200413172 | T | 0.6267 | -0.0314 |
| rs72923454 | T | 0.024 | 0.1164 |
| rs259848 | T | 0.6027 | 0.0288 |
| rs6433958 | T | 0.4957 | 0.0322 |
| rs13426118 | A | 0.8787 | -0.0578 |
| rs1047891 | A | 0.3168 | -0.0388 |
| rs6745160 | T | 0.4763 | 0.0299 |
| rs2943656 | A | 0.3666 | -0.0285 |
| rs11709077 | A | 0.1221 | -0.0579 |
| rs13069238 | A | 0.6005 | -0.0372 |
| rs10049237 | T | 0.2421 | -0.0378 |
| rs79672978 | C | 0.0903 | 0.0556 |
| rs139125439 | A | 0.8984 | 0.0517 |
| rs9847710 | T | 0.5825 | -0.0917 |
| rs13085838 | A | 0.3064 | -0.0337 |
| rs62252461 | A | 0.3558 | -0.0327 |
| rs1274226 | T | 0.2395 | -0.0365 |
| rs6438995 | A | 0.2007 | -0.0520 |
| rs76651220 | A | 0.0508 | 0.0986 |
| rs6769236 | C | 0.65 | -0.0431 |
| rs2862125 | T | 0.8033 | 0.0357 |
| rs112931950 | A | 0.9635 | 0.0806 |
| rs565519865 | T | 0.9296 | 0.0631 |
| rs62294340 | A | 0.3686 | -0.0392 |
| rs35297160 | C | 0.878 | -0.0522 |
| rs142552223 | A | 0.0382 | 0.0861 |
| rs9880232 | A | 0.7032 | -0.0322 |
| rs11723654 | A | 0.5292 | -0.0364 |
| rs7675964 | T | 0.2804 | -0.3970 |
| rs28407119 | T | 0.5703 | 0.0336 |
| rs11940694 | A | 0.4106 | -0.0358 |
| rs77454139 | C | 0.3777 | 0.0345 |
| rs62298617 | A | 0.5324 | -0.0306 |
| rs1458038 | T | 0.2898 | -0.0403 |
| rs2231142 | T | 0.1097 | 0.5510 |
| rs29001570 | T | 0.986 | -0.1926 |
| rs6853633 | A | 0.0936 | 0.0647 |
| rs2850387 | A | 0.6029 | 0.0298 |
| rs2903386 | T | 0.6138 | -0.0355 |
| rs10518306 | T | 0.1177 | 0.0424 |
| rs7685464 | C | 0.327 | 0.0422 |
| rs7681300 | A | 0.0202 | -0.1336 |
| rs11930385 | T | 0.4058 | -0.0395 |
| rs13157326 | A | 0.4724 | 0.0274 |
| rs461660 | A | 0.5474 | 0.0446 |
| rs34525658 | A | 0.1653 | 0.0375 |
| rs12153205 | T | 0.8244 | -0.0412 |
| rs3776694 | A | 0.8061 | 0.0369 |
| rs62355987 | T | 0.8975 | -0.0543 |
| rs2560449 | A | 0.513 | 0.0308 |
| rs17632159 | C | 0.3035 | -0.0766 |
| rs7705189 | A | 0.5369 | -0.0426 |
| rs2282804 | A | 0.1236 | 0.0416 |
| rs75049939 | T | 0.6929 | 0.0302 |
| rs116402366 | A | 0.0256 | 0.1233 |
| rs115986297 | A | 0.453 | 0.0275 |
| rs1294437 | T | 0.3463 | 0.0358 |
| rs12211604 | A | 0.6272 | -0.0866 |
| rs4715379 | C | 0.2415 | -0.0366 |
| rs1359232 | A | 0.451 | -0.1540 |
| rs2395180 | T | 0.7142 | -0.0301 |
| rs241436 | A | 0.5285 | -0.0298 |
| rs3097671 | C | 0.165 | 0.0384 |
| rs6920453 | T | 0.2056 | 0.0350 |
| rs2815059 | A | 0.2926 | 0.0340 |
| rs4714677 | T | 0.5946 | -0.0491 |
| rs9472135 | T | 0.7023 | 0.0594 |
| rs2817419 | A | 0.7314 | 0.0460 |
| rs72868889 | T | 0.0605 | 0.0607 |
| rs7742431 | A | 0.4979 | -0.0274 |
| rs76717437 | A | 0.6686 | -0.0315 |
| rs6934962 | T | 0.4145 | 0.0425 |
| rs10782230 | A | 0.4861 | 0.0456 |
| rs2270089 | A | 0.9027 | -0.0455 |
| rs3012413 | T | 0.1741 | -0.0382 |
| rs7785293 | T | 0.2468 | -0.0561 |
| rs11770600 | C | 0.1398 | -0.0441 |
| rs2721779 | A | 0.6484 | 0.0318 |
| rs1635852 | T | 0.4985 | 0.0299 |
| rs12673242 | T | 0.8528 | -0.0421 |
| rs56200772 | C | 0.9111 | 0.0631 |
| rs38305 | T | 0.4931 | -0.0300 |
| rs9955 | T | 0.1238 | -0.0597 |
| rs13246993 | A | 0.1253 | -0.1260 |
| rs12112658 | T | 0.7925 | 0.0354 |
| rs112758337 | A | 0.1776 | -0.0525 |
| rs45446698 | T | 0.9652 | 0.0950 |
| rs4727501 | A | 0.4373 | -0.0277 |
| rs4728141 | T | 0.5272 | -0.0306 |
| rs157934 | T | 0.7034 | 0.0382 |
| rs10487888 | T | 0.5287 | 0.0295 |
| rs148190310 | A | 0.0224 | 0.1121 |
| rs6977081 | T | 0.3462 | 0.0317 |
| rs10224210 | T | 0.712 | -0.0615 |
| rs7012637 | A | 0.4717 | 0.0318 |
| rs444480 | A | 0.6175 | 0.0362 |
| rs2466077 | T | 0.544 | -0.0393 |
| rs4537271 | A | 0.7614 | 0.0336 |
| rs1515020 | T | 0.4751 | 0.0545 |
| rs9297945 | A | 0.6283 | -0.0316 |
| rs2511656 | T | 0.1405 | 0.0510 |
| rs2737212 | T | 0.5647 | -0.0335 |
| rs62523889 | T | 0.4836 | -0.0311 |
| rs7845216 | A | 0.1931 | -0.0457 |
| rs12376901 | C | 0.2151 | -0.0419 |
| rs3824458 | T | 0.3181 | -0.0441 |
| rs10972072 | A | 0.7763 | -0.0327 |
| rs1412987 | A | 0.6744 | 0.0326 |
| rs10820911 | T | 0.3038 | -0.0307 |
| rs10761194 | A | 0.4893 | 0.0306 |
| rs2437817 | A | 0.3523 | -0.0386 |
| rs10987826 | C | 0.0518 | -0.0861 |
| rs7861143 | A | 0.4604 | 0.0291 |
| rs550057 | T | 0.2607 | -0.0339 |
| rs3750508 | T | 0.3679 | 0.0297 |
| rs73592376 | T | 0.1049 | 0.0662 |
| rs10994860 | T | 0.1803 | 0.0900 |
| rs12569559 | T | 0.5283 | 0.0689 |
| rs1171614 | T | 0.2253 | -0.1218 |
| rs2236295 | T | 0.3913 | -0.0369 |
| rs10740125 | T | 0.5143 | 0.0497 |
| rs10823237 | C | 0.3681 | 0.0285 |
| rs60783257 | T | 0.6182 | -0.0563 |
| rs6584390 | T | 0.7237 | -0.0367 |
| rs7093087 | A | 0.181 | 0.0431 |
| rs12251238 | T | 0.2786 | -0.0416 |
| rs181669 | T | 0.4514 | -0.0424 |
| rs10886117 | A | 0.1623 | 0.0586 |
| rs10794177 | C | 0.2571 | 0.0491 |
| rs12762637 | T | 0.7954 | 0.0412 |
| rs61063671 | A | 0.0969 | -0.0493 |
| rs11042549 | A | 0.4914 | -0.0284 |
| rs61888762 | C | 0.6961 | -0.0368 |
| rs10501093 | A | 0.1245 | -0.0425 |
| rs490937 | T | 0.687 | -0.0410 |
| rs3925584 | T | 0.5453 | 0.0540 |
| rs6484504 | T | 0.2697 | 0.0329 |
| rs499318 | A | 0.6567 | 0.0292 |
| rs10750861 | T | 0.6108 | 0.0294 |
| rs61897795 | A | 0.8524 | -0.0416 |
| rs150920552 | A | 0.0042 | 0.2483 |
| rs12363578 | T | 0.4164 | -0.1515 |
| rs642803 | T | 0.4564 | -0.0983 |
| rs117374064 | T | 0.0083 | 0.1582 |
| rs1789166 | A | 0.6531 | 0.0386 |
| rs10899113 | A | 0.0996 | 0.0555 |
| rs2513998 | C | 0.2596 | -0.0339 |
| rs631318 | A | 0.3628 | 0.0326 |
| rs573396 | A | 0.5333 | -0.0323 |
| rs896693 | A | 0.4322 | -0.0328 |
| rs4765929 | T | 0.6313 | -0.0291 |
| rs117233107 | A | 0.0158 | -0.1323 |
| rs58310495 | T | 0.1631 | -0.0497 |
| rs11181466 | A | 0.607 | 0.0379 |
| rs7132908 | A | 0.3917 | 0.0287 |
| rs12230839 | A | 0.7021 | -0.0400 |
| rs117672487 | A | 0.0517 | 0.0836 |
| rs2071450 | T | 0.3679 | -0.0293 |
| rs2638315 | C | 0.187 | 0.0370 |
| rs7964492 | A | 0.7737 | 0.1178 |
| rs12825843 | T | 0.3299 | -0.0338 |
| rs111391193 | A | 0.824 | 0.0453 |
| rs4766578 | A | 0.4936 | -0.0538 |
| rs1800574 | T | 0.0294 | -0.0870 |
| rs28707337 | T | 0.518 | 0.0546 |
| rs75312918 | A | 0.1439 | 0.0609 |
| rs1360485 | T | 0.7072 | 0.0331 |
| rs78375967 | C | 0.8181 | 0.0367 |
| rs9318029 | T | 0.2908 | -0.0315 |
| rs78477512 | T | 0.0231 | 0.1029 |
| rs6491286 | T | 0.5361 | -0.0300 |
| rs7139937 | T | 0.8563 | -0.0403 |
| rs72683923 | T | 0.9813 | 0.1421 |
| rs11623662 | T | 0.3819 | 0.0308 |
| rs11621358 | A | 0.1652 | -0.0438 |
| rs72706475 | A | 0.2874 | 0.0305 |
| rs11160674 | T | 0.8925 | -0.0517 |
| rs709400 | A | 0.6229 | 0.0352 |
| rs2412412 | A | 0.7103 | -0.0307 |
| rs12912771 | A | 0.247 | -0.0404 |
| rs4777542 | T | 0.3198 | -0.0310 |
| rs112752532 | T | 0.6041 | -0.0290 |
| rs10851885 | A | 0.7538 | -0.0850 |
| rs76871672 | T | 0.0597 | 0.0636 |
| rs12913266 | A | 0.3985 | 0.0312 |
| rs8024386 | A | 0.7555 | -0.0519 |
| rs7166287 | T | 0.6339 | -0.0582 |
| rs75712687 | A | 0.1702 | 0.0408 |
| rs879620 | T | 0.6092 | 0.0311 |
| rs30221 | T | 0.5423 | 0.0283 |
| rs13329952 | T | 0.8072 | 0.0387 |
| rs111873081 | T | 0.0731 | 0.0654 |
| rs11075990 | A | 0.5981 | -0.0573 |
| rs9980 | C | 0.8596 | -0.0980 |
| rs8046629 | A | 0.2922 | 0.0339 |
| rs73575079 | A | 0.6845 | 0.0575 |
| rs58323452 | A | 0.8206 | -0.0669 |
| rs16965913 | T | 0.3492 | 0.0303 |
| rs58912472 | A | 0.4846 | -0.0336 |
| rs11078711 | T | 0.0726 | 0.0691 |
| rs2428365 | A | 0.3919 | 0.0311 |
| rs2453579 | A | 0.4306 | -0.0323 |
| rs9896098 | A | 0.0901 | 0.0585 |
| rs56336338 | A | 0.1361 | 0.0439 |
| rs28394864 | A | 0.4664 | 0.0365 |
| rs7224610 | A | 0.5893 | -0.0535 |
| rs1292041 | A | 0.4681 | -0.0339 |
| rs2378816 | A | 0.8025 | -0.0546 |
| rs9905274 | T | 0.1811 | -0.0703 |
| rs77542162 | A | 0.9816 | 0.1999 |
| rs164011 | A | 0.3224 | -0.0344 |
| rs103550 | C | 0.686 | -0.0297 |
| rs9894993 | T | 0.5419 | 0.0283 |
| rs489837 | T | 0.5543 | 0.0284 |
| rs17740231 | A | 0.2975 | 0.0295 |
| rs2046243 | A | 0.2418 | 0.0323 |
| rs538656 | T | 0.2331 | 0.0624 |
| rs78274649 | A | 0.0184 | -0.1128 |
| rs10405423 | A | 0.6638 | 0.0475 |
| rs62126382 | T | 0.4991 | -0.0420 |
| rs273496 | T | 0.3941 | 0.0363 |
| rs8106047 | A | 0.1618 | 0.0480 |
| rs12976336 | A | 0.4019 | -0.0426 |
| rs117035602 | A | 0.0474 | 0.0705 |
| rs739320 | T | 0.3763 | 0.0335 |
| rs190676167 | A | 0.0017 | 0.6336 |
| rs10421230 | A | 0.0264 | 0.1013 |
| rs2235811 | A | 0.5124 | 0.0291 |
| rs6058108 | A | 0.5974 | -0.0300 |
| rs6031601 | A | 0.6124 | -0.0382 |
| rs1689059 | A | 0.5647 | -0.0276 |
| rs9647055 | A | 0.1836 | 0.0385 |
| rs219787 | T | 0.2528 | -0.0469 |
| rs4817983 | C | 0.2723 | -0.0442 |
| rs4818067 | T | 0.7202 | -0.0332 |
| rs5762683 | T | 0.5115 | -0.0345 |
| rs146173943 | A | 0.0708 | -0.0719 |
| rs738408 | T | 0.2238 | -0.0828 |
| rs7876156 | T | 0.2381 | -0.0321 |
| rs57434549 | T | 0.3789 | 0.0251 |
| rs1802288 | T | 0.1758 | 0.0424 |
| rs17003845 | A | 0.2109 | 0.0311 |
| rs432528 | C | 0.7567 | 0.0325 |
| rs7061622 | T | 0.5955 | 0.0323 |
| rs41312616 | T | 0.037 | 0.0631 |
| rs4262465 | A | 0.5901 | 0.0341 |
| **Low back pain** |  |  |  |
| rs55692411 | G | 0.5736 | -0.03067 |
| rs13107325 | C | 0.9167 | -0.07077 |
| rs12513045 | T | 0.6561 | 0.03072 |
| rs9687387 | T | 0.6852 | -0.03229 |
| rs112540634 | C | 0.8533 | -0.04001 |
| rs12538901 | T | 0.2868 | 0.03328 |
| rs10280045 | C | 0.4414 | -0.03624 |
| rs11562624 | G | 0.3617 | 0.03701 |
| rs12268174 | T | 0.506 | -0.0446 |
| rs3896224 | A | 0.594 | 0.03038 |
| rs34369393 | G | 0.4419 | -0.02982 |
| rs4245150 | G | 0.3704 | -0.02966 |
| rs12901499 | G | 0.5481 | 0.03122 |
| rs564957276 | C | 0.9603 | 0.09909 |
| **Neck pain** |  |  |  |
| rs159963 | C | 0.4511 | 0.04301 |
| rs6974757 | C | 0.4254 | -0.03823 |

| **Table S7. Detailed information on covariates.** | | |
| --- | --- | --- |
| **Covariates** | **Field ID** | **Descriptions** |
| Age | 21003 | continuous, years |
| Sex | 31 | Female, Male |
| Assessment center | 54 | England, Scotland, Wales |
| Education level | 6138 | college or university degree, non-college or university degree |
| Current employment status | 6142 | employed, unemployed |
| Townsend Deprivation Index | 22189 | continuous; a higher score indicates a higher degree of deprivation |
| Grip strength | 46 and 47 | continuous, kg |
| SBP (systolic blood pressure) | 4080 and 93 | continuous, mmHg |
| DBP (diastolic blood pressure) | 4079 and 94 | continuous, mmHg |
| Antihypertensive medication use | 6153 and 6177 | yes, no |
| Antidiabetic medication use | 6153 and 6177 | yes, no |
| Statin use | 6153 and 6177 | yes, no |
| Smoking status | 20116 | never, previous, current |
| Alcohol consumption status | 20117 | never, previous, current |
| Physical activity | 22040 | continuous, minutes, metabolic equivalent task—sum of minutes performing walking, moderate, and vigorous activity |
| Sleep duration | 1160 | short: ≤6 hours, day; normal: 7 to 8 hours, day; and long: ≥9 hours, day |
| Healthy diet score | 1289/1299/1309/1319/1329/1339/1349/1369/1379/1389 | continuous, was calculated by using the following dietary factors: vegetable intake at least four tablespoons each day (median); fruits intake at least three pieces each day (median); fish intake at least twice each week (median); unprocessed red meat intake no more than twice each week (median); and processed meat intake no more than two each week (median). Each one point was given for each favorable diet factor, with the total diet score ranging from 0 to 5. |
| BMI (body mass index) | 21001 | continuous, in kg/m2 |
| Top 10 genetic principal components | 22009 | used in analyses involving genetic data |
| Genotype batch | 22000 | used in analyses involving genetic data |

| **Table S8. Baseline characteristics of participants in the UK Biobank by social isolation status.** | | | | |
| --- | --- | --- | --- | --- |
| **Baseline characteristics** | **Total** | **Not isolated** | **Isolated** | P value |
| **No. of participants** | 315,197 | 288,539 | 26,658 |  |
| **Age, years** | 55.9 ± 8.1 | 55.9 ± 8.1 | 56.1 ± 7.8 | < 0.001 |
| **Male, n (%)** | 146,126 (46.4) | 132,528 (45.9) | 13,598 (51.0) | < 0.001 |
| **Assessment center** |  |  |  | < 0.001 |
| England, n (%) | 276,834 (87.8) | 253,283 (87.8) | 23,551 (88.3) |  |
| Scotland, n (%) | 24,342 (7.7) | 22,274 (7.7) | 2,068 (7.8) |  |
| Wales, n (%) | 14,021 (4.4) | 12,982 (4.5) | 1,039 (3.9) |  |
| **College/university degree, n (%)** | 110,961 (35.2) | 102,203 (35.4) | 8,758 (32.9) | < 0.001 |
| **Employed, n (%)** | 196,820 (62.4) | 180,116 (62.4) | 16,704 (62.7) | < 0.001 |
| **TDI** | -1.6 ± 2.9 | -1.7 ± 2.8 | -0.3 ± 3.4 | < 0.001 |
| **Grip strength, kg** | 31.6 ± 10.7 | 31.6 ± 10.7 | 31.3 ± 10.6 | < 0.001 |
| **SBP, mmHg** | 137.4 ± 18.7 | 137.4 ± 18.7 | 137.7 ± 18.7 | 0.006 |
| **DBP, mmHg** | 82.1 ± 10.2 | 82.1 ± 10.1 | 82.6 ± 10.5 | < 0.001 |
| **Antihypertensive medication use, n (%)** | 56,681 (18.0) | 51,180 (17.7) | 5,501 (20.6) | < 0.001 |
| **Antidiabetic medication use, n (%)** | 2,727 (0.9) | 2,367 (0.8) | 360 (1.4) | < 0.001 |
| **Statin use, n (%)** | 47,244 (15.0) | 42,646 (14.8) | 4,598 (17.2) | < 0.001 |
| **Current smoker, n (%)** | 31,604 (10.0) | 26,873 (9.3) | 4,731 (17.7) | < 0.001 |
| **Current drinker, n (%)** | 296,839 (94.2) | 273,045 (94.6) | 23,794 (89.3) | < 0.001 |
| **Physical activity, MET-min/week** | 2,637.0 ± 2,609.9 | 2662.6 ± 2600.3 | 2349.1 ± 2699.7 | < 0.001 |
| **Sleep duration** |  |  |  | < 0.001 |
| Normal, 7-8 h/d, n (%) | 222,155 (70.5) | 205,509 (71.2) | 16,646 (62.4) |  |
| Short, <7 h/d, n (%) | 69,870 (22.2) | 62,149 (21.5) | 7,721 (29.0) |  |
| Long, >8 h/d, n (%) | 22,190 (7.0) | 20,086 (7.0) | 2,104 (7.9) |  |
| **Healthy diet score** | 3.0 (2.0, 4.0) | 3.0 (2.0, 4.0) | 3.0 (2.0, 4.0) | < 0.001 |
| **BMI** | 27.0 ± 4.4 | 26.9 ± 4.3 | 27.3 ± 4.8 | < 0.001 |
| Abbreviations: TDI, Townsend deprivation index; SBP, systolic blood pressure; DBP, diastolic blood pressure; MET, metabolic equivalent of task; BMI, body mass index. Healthy diet score: Range 0-5 points; positively linked with the level of adherence to a healthy diet. | | | | |

| **Table S9. Associations of the scores and items of loneliness with long-term risk of musculoskeletal disorders.** | | | | |
| --- | --- | --- | --- | --- |
|  | **N** | **Cases/Person-years** | **Model 1 HR (95% CI)** | **Model 2 HR (95% CI)** |
| **Overall MSK** | | | | |
| Loneliness scores | | | | |
| 0 point | 219,589 | 53,459/2,614,524.6 | 1.00 Ref. | 1.00 Ref. |
| 1 point | 77,866 | 21,040/908,766.1 | **1.13 (1.11-1.15)** | **1.07 (1.06-1.09)** |
| 2 points | 17,742 | 5,190/203,250.6 | **1.30 (1.26-1.34)** | **1.16 (1.13-1.20)** |
| P trend |  |  | **< 0.001** | **< 0.001** |
| Items of loneliness | | | | |
| Feeling lonely | | | | |
| No | 262,529 | 64,809/3,117,078.7 | 1.00 Ref. | 1.00 Ref. |
| Yes | 52,668 | 14,880/609,462.6 | **1.24 (1.22-1.27)** | **1.14 (1.12-1.16)** |
| Willing to confide | | | | |
| Yes | 254,515 | 63,149/3,020,736.7 | 1.00 Ref. | 1.00 Ref. |
| No | 60,682 | 16,540/705,804.6 | **1.09 (1.07-1.11)** | **1.04 (1.03-1.06)** |
| **OA** | | | | |
| Loneliness scores | | | | |
| 0 point | 219,589 | 24,736/2,800,099.8 | 1.00 Ref. | 1.00 Ref. |
| 1 point | 77,866 | 9,698/982,787.9 | **1.11 (1.08-1.13)** | **1.05 (1.03-1.08)** |
| 2 points | 17,742 | 2,320/222,150.1 | **1.24 (1.19-1.29)** | **1.10 (1.06-1.15)** |
| P trend |  |  | **< 0.001** | **< 0.001** |
| Items of loneliness | | | | |
| Feeling lonely | | | | |
| No | 262,529 | 30,040/3,341,332.2 | 1.00 Ref. | 1.00 Ref. |
| Yes | 52,668 | 6,714/663,705.6 | **1.21 (1.18-1.24)** | **1.10 (1.07-1.13)** |
| Willing to confide | | | | |
| Yes | 254,515 | 29,130/3,241,655.4 | 1.00 Ref. | 1.00 Ref. |
| No | 60,682 | 7,624/763,382.4 | **1.07 (1.04-1.10)** | 1.02 (1.00-1.05) |
| **Gout** | | | | |
| Loneliness scores | | | | |
| 0 point | 219,589 | 2,521/2,929,898.0 | 1.00 Ref. | 1.00 Ref. |
| 1 point | 77,866 | 1,066/1,032,586.0 | **1.16 (1.08-1.25)** | 1.07 (1.00-1.15) |
| 2 points | 17,742 | 277/234,281.0 | **1.43 (1.26-1.62)** | **1.16 (1.02-1.31)** |
| P trend |  |  | **< 0.001** | **0.007** |
| Items of loneliness | | | | |
| Feeling lonely | | | | |
| No | 262,529 | 3,241/3,497,879.1 | 1.00 Ref. | 1.00 Ref. |
| Yes | 52,668 | 623/698,885.7 | **1.28 (1.17-1.39)** | 1.07 (0.98-1.17) |
| Willing to confide | | | | |
| Yes | 254,515 | 2,867/3,394,502.4 | 1.00 Ref. | 1.00 Ref. |
| No | 60,682 | 997/802,262.4 | **1.18 (1.09-1.26)** | **1.10 (1.02-1.18)** |
| **RA** | | | | |
| Loneliness scores | | | | |
| 0 point | 219,589 | 1,571/2,932,517.6 | 1.00 Ref. | 1.00 Ref. |
| 1 point | 77,866 | 691/1,033,620.2 | **1.24 (1.14-1.36)** | **1.14 (1.04-1.24)** |
| 2 points | 17,742 | 181/234,437.9 | **1.51 (1.30-1.76)** | **1.25 (1.07-1.46)** |
| P trend |  |  | **< 0.001** | **< 0.001** |
| Items of loneliness | | | | |
| Feeling lonely | | | | |
| No | 262,529 | 1,930/3,501,693.9 | 1.00 Ref. | 1.00 Ref. |
| Yes | 52,668 | 513/698,881.9 | **1.39 (1.26-1.53)** | **1.19 (1.08-1.32)** |
| Willing to confide | | | | |
| Yes | 254,515 | 1,903/3,396,961.5 | 1.00 Ref. | 1.00 Ref. |
| No | 60,682 | 540/803,614.2 | **1.19 (1.08-1.31)** | **1.11 (1.00-1.22)** |
| **Low back pain** | | | | |
| Loneliness scores | | | | |
| 0 point | 219,589 | 4,062/2,919,451.2 | 1.00 Ref. | 1.00 Ref. |
| 1 point | 77,866 | 1,749/1,027,971.2 | **1.22 (1.15-1.29)** | **1.11 (1.05-1.17)** |
| 2 points | 17,742 | 530/232,594.7 | **1.68 (1.54-1.84)** | **1.36 (1.24-1.49)** |
| P trend |  |  | **< 0.001** | **< 0.001** |
| Items of loneliness | | | | |
| Feeling lonely | | | | |
| No | 262,529 | 4,943/3,486,005.1 | 1.00 Ref. | 1.00 Ref. |
| Yes | 52,668 | 1,398/694,011.9 | **1.48 (1.40-1.57)** | **1.26 (1.19-1.34)** |
| Willing to confide | | | | |
| Yes | 254,515 | 4,930/3,380,868.4 | 1.00 Ref. | 1.00 Ref. |
| No | 60,682 | 1,411/799,148.6 | **1.19 (1.12-1.26)** | **1.09 (1.03-1.16)** |
| **Neck pain** | | | | |
| Loneliness scores | | | | |
| 0 point | 219,589 | 822/2,935,530.5 | 1.00 Ref. | 1.00 Ref. |
| 1 point | 77,866 | 381/1,034,884.4 | **1.31 (1.16-1.48)** | **1.19 (1.05-1.35)** |
| 2 points | 17,742 | 110/234,692.6 | **1.71 (1.40-2.08)** | **1.37 (1.12-1.68)** |
| P trend |  |  | **< 0.001** | **< 0.001** |
| Items of loneliness | | | | |
| Feeling lonely | | | | |
| No | 262,529 | 987/3,505,587.3 | 1.00 Ref. | 1.00 Ref. |
| Yes | 52,668 | 326/699,520.3 | **1.70 (1.49-1.92)** | **1.45 (1.27-1.64)** |
| Willing to confide | | | | |
| Yes | 254,515 | 1,038/3,400,358.2 | 1.00 Ref. | 1.00 Ref. |
| No | 60,682 | 275/804,749.4 | 1.12 (0.98-1.27) | 1.02 (0.89-1.16) |
| Model 1: adjusted for age and sex. Model 2: additionally adjusted for assessment center, education level, current employment status, Townsend deprivation index, grip strength, systolic blood pressure, diastolic blood pressure, antihypertensive medication use, antidiabetic medication use, statin use, smoking status, alcohol consumption status, physical activity, sleep duration, healthy diet score and BMI, based on model 1. Abbreviations: MSK, musculoskeletal disorders; OA, osteoarthritis; RA, rheumatoid arthritis; HR, hazard ratio; CI, confidence interval. | | | | |

| **Table S10. Associations of social isolation with subsequent risk for musculoskeletal disorders.** | | | | |
| --- | --- | --- | --- | --- |
|  | **N** | **Cases/Person-years** | **Model 1 HR (95% CI)** | **Model 2 HR (95% CI)** |
|  |  |  |  |  |
| **Overall MSK** | | | | |
| Not isolated | 288,539 | 72,923/3,417,926.8 | 1.00 Ref. | 1.00 Ref. |
| Isolated | 26,658 | 6,766/308,614.5 | **1.04 (1.01-1.06)** | **0.96 (0.94-0.99)** |
| **OA** | | | | |
| Not isolated | 288,539 | 33,861/3,672,235.0 | 1.00 Ref. | 1.00 Ref. |
| Isolated | 26,658 | 2,893/332,803.0 | **0.95 (0.92-0.99)** | **0.89 (0.86-0.93)** |
| **Gout** | | | | |
| Not isolated | 288,539 | 3,518/3,849,666.9 | 1.00 Ref. | 1.00 Ref. |
| Isolated | 26,658 | 346/347,097.9 | 1.07 (0.96-1.19) | 0.93 (0.83-1.04) |
| **RA** | | | | |
| Not isolated | 288,539 | 2,196/3,853,403.0 | 1.00 Ref. | 1.00 Ref. |
| Isolated | 26,658 | 247/347,172.7 | **1.28 (1.12-1.46)** | 1.10 (0.96-1.25) |
| **Low back pain** | | | | |
| Not isolated | 288,539 | 5,729/3,834,582.0 | 1.00 Ref. | 1.00 Ref. |
| Isolated | 26,658 | 612/345,435.0 | **1.20 (1.10-1.30)** | 1.01 (0.93-1.11) |
| **Neck pain** | | | | |
| Not isolated | 288,539 | 1,187/3,857,312.5 | 1.00 Ref. | 1.00 Ref. |
| Isolated | 26,658 | 126/347,795.1 | 1.19 (0.99-1.43) | 1.00 (0.83-1.21) |
| Model 1: adjusted for age and sex. Model 2: additionally adjusted for assessment center, education level, current employment status, Townsend deprivation index, grip strength, systolic blood pressure, diastolic blood pressure, antihypertensive medication use, antidiabetic medication use, statin use, smoking status, alcohol consumption status, physical activity, sleep duration, healthy diet score and BMI, based on model 1. Abbreviations: MSK, musculoskeletal disorders; OA, osteoarthritis; RA, rheumatoid arthritis; HR, hazard ratio; CI, confidence interval. | | | | |

| **Table S11. Relative importance of loneliness and traditional risk factors for predicting musculoskeletal disorders, by estimation of explained log-likelihood explained by each predictor.** | | | | | | | | | | | |
| --- | --- | --- | --- | --- | --- | --- | --- | --- | --- | --- | --- |
| **MSK** | | **OA** | | **Gout** | | **RA** | | **Low back pain** | | **Neck pain** | |
| BMI (3) | 0.5185 | BMI (3) | 0.7377 | BMI (3) | 0.8286 | BMI (3) | 0.2505 | BMI (3) | 0.3877 | **Loneliness (2)** | 0.2956 |
| Sleep (2) | 0.1763 | Sleep (2) | 0.0938 | Diet (5) | 0.0878 | Sleep (2) | 0.2386 | Sleep (2) | 0.1680 | Smoking (1) | 0.1716 |
| PA (2) | 0.1129 | PA (2) | 0.0762 | Sleep (2) | 0.0478 | Smoking (1) | 0.1568 | **Loneliness (2)** | 0.1582 | Sleep (2) | 0.1562 |
| **Loneliness (2)** | 0.0839 | Diet (5) | 0.0482 | **Loneliness (2)** | 0.0185 | **Loneliness (2)** | 0.1356 | Smoking (1) | 0.1388 | PA (2) | 0.1419 |
| Diet (5) | 0.0606 | **Loneliness (2)** | 0.0275 | PA (2) | 0.0121 | Alcohol (1) | 0.1039 | PA (2) | 0.0736 | BMI (3) | 0.1184 |
| Alcohol (1) | 0.0388 | Alcohol (1) | 0.0155 | Smoking (1) | 0.0027 | PA (2) | 0.0868 | Alcohol (1) | 0.0658 | Alcohol (1) | 0.0778 |
| Smoking (1) | 0.0090 | Smoking (1) | 0.0011 | Alcohol (1) | 0.0026 | Diet (5) | 0.0279 | Diet (5) | 0.0079 | Diet (5) | 0.0385 |
| Analysis was restricted to 250,372 participants who had complete data on loneliness and risk factor control. Relative variable importance was measured by means of explained log-likelihood for each predictor. The values in the table represent each predictor's chi-square contribution as a proportion of the total chi-square. The number presented in the parenthesis next to each predictor is the number of degrees of freedom used for each predictor. Abbreviations: MSK, musculoskeletal disorders; OA, osteoarthritis; RA, rheumatoid arthritis; PA, physical activity. | | | | | | | | | | | |

| **Table S12. Joint association and stratified analysis of loneliness and the degree of PRS on the risk of musculoskeletal disorders.** | | | | | | | |
| --- | --- | --- | --- | --- | --- | --- | --- |
| **Loneliness** | **PRS** | **Cases/Person-years** | **Joint effects** | | **Stratified analysis** | | |
|  |  |  | **HR (95% CI)** | P **value** | **HR (95% CI)** | P **value** | P **interaction** |
| **Overall MSK** | | | | | | | |
| No | Low | 11,309/579,846.5 | 1.00 Ref. | Ref. | 1.00 Ref. | Ref. | 0.626 |
| Yes | Low | 804/33,306.0 | **1.18 (1.09-1.26)** | **<.001** | **1.17 (1.09-1.26)** | **<.001** |  |
| No | Intermediate | 35,878/1,728,157.0 | **1.06 (1.04-1.08)** | **<.001** | 1.00 Ref. | Ref. |  |
| Yes | Intermediate | 2,537/99,922.9 | **1.22 (1.17-1.28)** | **<.001** | **1.15 (1.10-1.20)** | **<.001** |  |
| No | High | 12,961/569,763.2 | **1.16 (1.13-1.19)** | **<.001** | 1.00 Ref. | Ref. |  |
| Yes | High | 887/32,753.7 | **1.30 (1.22-1.40)** | **<.001** | **1.13 (1.05-1.21)** | **0.001** |  |
| **OA** | | | | | | | |
| No | Low | 4,966/621,330.0 | 1.00 Ref. | Ref. | 1.00 Ref. | Ref. | 0.186 |
| Yes | Low | 324/36,965.2 | 1.02 (0.91-1.14) | 0.692 | 1.01 (0.90-1.13) | 0.836 |  |
| No | Intermediate | 16,511/1,854,844.0 | **1.12 (1.09-1.16)** | **<.001** | 1.00 Ref. | Ref. |  |
| Yes | Intermediate | 1,157/109,313.8 | **1.27 (1.19-1.35)** | **<.001** | **1.13 (1.06-1.20)** | **<.001** |  |
| No | High | 6,229/614,561.5 | **1.29 (1.24-1.34)** | **<.001** | 1.00 Ref. | Ref. |  |
| Yes | High | 408/35,520.9 | **1.36 (1.23-1.50)** | **<.001** | 1.07 (0.97-1.19) | 0.171 |  |
| **Gout** | | | | | | | |
| No | Low | 235/648,671.6 | 1.00 Ref. | Ref. | 1.00 Ref. | Ref. | 0.363 |
| Yes | Low | 14/37,902.7 | 0.90 (0.53-1.55) | 0.706 | 0.87 (0.50-1.50) | 0.619 |  |
| No | Intermediate | 1,606/1,940,371.0 | **2.23 (1.95-2.56)** | **<.001** | 1.00 Ref. | Ref. |  |
| Yes | Intermediate | 121/115,772.7 | **2.41 (1.93-3.00)** | **<.001** | 1.07 (0.89-1.29) | 0.469 |  |
| No | High | 1,082/645,756.5 | **4.65 (4.03-5.35)** | **<.001** | 1.00 Ref. | Ref. |  |
| Yes | High | 90/37,913.7 | **5.89 (4.61-7.52)** | **<.001** | **1.28 (1.03-1.60)** | **0.025** |  |
| **RA** | | | | | | | |
| No | Low | 261/778,000.9 | 1.00 Ref. | Ref. | 1.00 Ref. | Ref. | 0.820 |
| Yes | Low | 22/44,998.9 | 1.29 (0.84-2.00) | 0.246 | 1.29 (0.83-2.01) | 0.250 |  |
| No | Intermediate | 1,233/2,329,191.0 | **1.57 (1.37-1.79)** | **<.001** | 1.00 Ref. | Ref. |  |
| Yes | Intermediate | 99/137,644.1 | **1.88 (1.49-2.37)** | **<.001** | 1.18 (0.96-1.45) | 0.114 |  |
| No | High | 706/774,633.7 | **2.66 (2.31-3.07)** | **<.001** | 1.00 Ref. | Ref. |  |
| Yes | High | 52/46,101.6 | **2.94 (2.18-3.97)** | **<.001** | 1.14 (0.86-1.51) | 0.374 |  |
| **Low back pain** | | | | | | | |
| No | Low | 857/644,954.4 | 1.00 Ref. | Ref. | 1.00 Ref. | Ref. | 0.744 |
| Yes | Low | 82/37,946.8 | **1.37 (1.09-1.71)** | **0.007** | **1.36 (1.08-1.71)** | **0.010** |  |
| No | Intermediate | 2,807/1,934,356.0 | **1.08 (1.00-1.17)** | **0.038** | 1.00 Ref. | Ref. |  |
| Yes | Intermediate | 262/113,650.5 | **1.47 (1.28-1.69)** | **<.001** | **1.37 (1.20-1.55)** | **<.001** |  |
| No | High | 1,050/643,508.0 | **1.21 (1.11-1.32)** | **<.001** | 1.00 Ref. | Ref. |  |
| Yes | High | 92/38,584.1 | **1.50 (1.21-1.86)** | **<.001** | 1.22 (0.98-1.51) | 0.072 |  |
| **Neck pain** | | | | | | | |
| No | Low | 288/1,013,245.0 | 1.00 Ref. | Ref. | 1.00 Ref. | Ref. | 0.501 |
| Yes | Low | 31/59,075.3 | **1.53 (1.05-2.22)** | **0.026** | **1.53 (1.05-2.24)** | **0.028** |  |
| No | Intermediate | 497/1,598,138.0 | 1.10 (0.95-1.27) | 0.210 | 1.00 Ref. | Ref. |  |
| Yes | Intermediate | 40/95,064.4 | 1.26 (0.90-1.76) | 0.176 | 1.14 (0.82-1.58) | 0.425 |  |
| No | High | 220/629,759.9 | **1.23 (1.03-1.47)** | **0.021** | 1.00 Ref. | Ref. |  |
| Yes | High | 19/37,766.6 | 1.47 (0.92-2.34) | 0.108 | 1.21 (0.75-1.94) | 0.439 |  |
| The analysis was performed in Model 2 (adjusted with age, sex, assessment center, education level, current employment status, Townsend deprivation index, grip strength, systolic blood pressure, diastolic blood pressure, antihypertensive medication use, antidiabetic medication use, statin use, smoking status, alcohol consumption status, physical activity, sleep duration, healthy diet score and BMI), with additional adjustment for genetic principal components (PCs) 1-10, genotyping array. Abbreviations: MSK, musculoskeletal disorders; PRS, polygenic risk score; BMI, body mass index; OA, osteoarthritis; RA, rheumatoid arthritis; HR, hazard ratio; CI, confidence interval. | | | | | | | |

| **Table S13. Interaction effects between loneliness and PRS on the risk of musculoskeletal disorders in the UKB cohort.** | | | | |
| --- | --- | --- | --- | --- |
|  | **Additive interaction** | | | **Multiplicative Interaction** |
|  | ***RERI* (95% CI)** | ***AP* (95% CI)** | ***S* (95% CI)** | **HR (95% CI)** |
| **Overall MSK** | 0.01 (-0.06, 0.09) | 0.01 (-0.05, 0.07) | 1.06 (0.78, 1.44) | 1.00 (0.94, 1.07) |
| **OA** | 0.03 (-0.08, 0.14) | 0.03 (-0.06, 0.11) | 1.14 (0.75, 1.73) | 1.02 (0.93, 1.12) |
| **Gout** | 0.38 (-0.12, 0.88) | 0.14 (-0.03, 0.31) | 1.28 (0.93, 1.77) | 1.16 (0.85, 1.57) |
| **RA** | 0.04 (-0.45, 0.52) | 0.02 (-0.23, 0.27) | 1.04 (0.61, 1.76) | 0.94 (0.69, 1.29) |
| **Low back pain** | -0.1 (-0.37, 0.17) | -0.07 (-0.27, 0.13) | 0.80 (0.43, 1.46) | 0.91 (0.75, 1.11) |
| **Neck pain** | -0.25 (-0.87, 0.38) | -0.19 (-0.68, 0.31) | 0.57 (0.14, 2.25) | 0.81 (0.52, 1.28) |
| The analysis was performed in Model 2 (adjusted with age, sex, assessment center, education level, current employment status, Townsend deprivation index, grip strength, systolic blood pressure, diastolic blood pressure, antihypertensive medication use, antidiabetic medication use, statin use, smoking status, alcohol consumption status, physical activity, sleep duration, healthy diet score and BMI), with additional adjustment for genetic principal components (PCs) 1-10, genotyping array. Abbreviations: MSK, musculoskeletal disorders; OA, osteoarthritis; RA, rheumatoid arthritis; HR, hazard ratio; CI, confidence interval; RERl, relative excess risk due to interaction; AP, attributable proportion due to interaction; S, the synergy index. | | | | |

| **Table S14. Multivariable-adjusted HRs (95% CI) of loneliness or social isolation for musculoskeletal disorders, after mutual adjustment in models included all covariates.** | | | | | | |
| --- | --- | --- | --- | --- | --- | --- |
|  | **Overall MSK** | **OA** | **Gout** | **RA** | **Low back pain** | **Neck pain** |
| **Loneliness** | | | | | | |
| No loneliness | 1.00 Ref. | 1.00 Ref. | 1.00 Ref. | 1.00 Ref. | 1.00 Ref. | 1.00 Ref. |
| Loneliness | **1.15 (1.11-1.18)** | **1.10 (1.06-1.15)** | **1.15 (1.01-1.30)** | **1.18 (1.02-1.38)** | **1.32 (1.21-1.45)** | **1.30 (1.06-1.59)** |
| **Social isolation** | | | | | | |
| No isolated | 1.00 Ref. | 1.00 Ref. | 1.00 Ref. | 1.00 Ref. | 1.00 Ref. | 1.00 Ref. |
| Isolated | **0.95 (0.93-0.98)** | **0.88 (0.85-0.92)** | 0.92 (0.82-1.03) | 1.08 (0.94-1.23) | 0.99 (0.91-1.07) | 0.98 (0.81-1.18) |
| HRs were adjusted for age, sex, assessment center, education level, current employment status, Townsend deprivation index, grip strength, systolic blood pressure, diastolic blood pressure, antihypertensive medication use, antidiabetic medication use, statin use, smoking status, alcohol consumption status, physical activity, sleep duration, healthy diet score, BMI, and social isolation or loneliness. Abbreviations: MSK, musculoskeletal disorders; OA, osteoarthritis; RA, rheumatoid arthritis; HR, hazard ratio; CI, confidence interval. | | | | | | |

| **Table S15. Associations of loneliness and social isolation with subsequent risk for musculoskeletal disorders after excluding those occurring musculoskeletal disorders within the first two years in the UKB cohort (n = 304,834).** | | | | |
| --- | --- | --- | --- | --- |
|  | **N** | **Cases/Person-years** | **Model 1 HR (95% CI)** | **Model 2 HR (95% CI)** |
|  |  |  |  |  |
| **Overall MSK** | | | | |
| **Loneliness** |  |  |  |  |
| No loneliness | 287,851 | 66,128/3,512,980.3 | 1.00 Ref. | 1.00 Ref. |
| Loneliness | 16,983 | 4,523/202,451.7 | **1.24 (1.20-1.28)** | **1.13 (1.09-1.16)** |
| **Social isolation** |  |  |  |  |
| No isolated | 279,208 | 64,698/3,407,886.2 | 1.00 Ref. | 1.00 Ref. |
| Isolated | 25,626 | 5,953/307,545.7 | 1.03 (1.00-1.06) | **0.96 (0.93-0.99)** |
| **OA** | | | | |
| **Loneliness** |  |  |  |  |
| No loneliness | 287,851 | 30,546/3,711,608.0 | 1.00 Ref. | 1.00 Ref. |
| Loneliness | 16,983 | 2,019/216,416.0 | **1.19 (1.14-1.25)** | **1.08 (1.03-1.13)** |
| **Social isolation** |  |  |  |  |
| No isolated | 279,208 | 30,009/3,602,350.7 | 1.00 Ref. | 1.00 Ref. |
| Isolated | 25,626 | 2,556/325,673.6 | **0.95 (0.92-0.99)** | **0.90 (0.86-0.93)** |
| **Gout** | | | | |
| **Loneliness** |  |  |  |  |
| No loneliness | 287,851 | 3,333/3,854,418.2 | 1.00 Ref. | 1.00 Ref. |
| Loneliness | 16,983 | 258/225,858.6 | **1.39 (1.22-1.58)** | **1.15 (1.01-1.31)** |
| **Social isolation** |  |  |  |  |
| No isolated | 279,208 | 3,267/3,743,255.6 | 1.00 Ref. | 1.00 Ref. |
| Isolated | 25,626 | 324/337,021.3 | 1.08 (0.96-1.21) | 0.93 (0.83-1.05) |
| **RA** | | | | |
| **Loneliness** |  |  |  |  |
| No loneliness | 287,851 | 2,045/3,858,189.0 | 1.00 Ref. | 1.00 Ref. |
| Loneliness | 16,983 | 159/226,076.0 | **1.39 (1.19-1.64)** | **1.19 (1.01-1.39)** |
| **Social isolation** |  |  |  |  |
| No isolated | 279,208 | 1,981/3,747,105.0 | 1.00 Ref. | 1.00 Ref. |
| Isolated | 25,626 | 223/337,160.3 | **1.28 (1.11-1.47)** | 1.11 (0.96-1.27) |
| **Low back pain** | | | | |
| **Loneliness** |  |  |  |  |
| No loneliness | 287,851 | 5,146/3,843,283.3 | 1.00 Ref. | 1.00 Ref. |
| Loneliness | 16,983 | 445/224,728.6 | **1.52 (1.38-1.68)** | **1.27 (1.15-1.40)** |
| **Social isolation** |  |  |  |  |
| No isolated | 279,208 | 5,059/3,732,186.8 | 1.00 Ref. | 1.00 Ref. |
| Isolated | 25,626 | 532/335,825.1 | **1.18 (1.08-1.29)** | 1.01 (0.92-1.10) |
| **Neck pain** | | | | |
| **Loneliness** |  |  |  |  |
| No loneliness | 287,851 | 1,084/3,861,720.9 | 1.00 Ref. | 1.00 Ref. |
| Loneliness | 16,983 | 89/226,300.4 | **1.43 (1.15-1.77)** | 1.18 (0.95-1.47) |
| **Social isolation** |  |  |  |  |
| No isolated | 279,208 | 1,065/3,750,320.8 | 1.00 Ref. | 1.00 Ref. |
| Isolated | 25,626 | 108/337,700.6 | 1.14 (0.93-1.39) | 0.98 (0.80-1.20) |
| Model 1: adjusted for age and sex. Model 2: additionally adjusted for assessment center, education level, current employment status, Townsend deprivation index, grip strength, systolic blood pressure, diastolic blood pressure, antihypertensive medication use, antidiabetic medication use, statin use, smoking status, alcohol consumption status, physical activity, sleep duration, healthy diet score and BMI, based on model 1. Abbreviations: MSK, musculoskeletal disorders; OA, osteoarthritis; RA, rheumatoid arthritis; HR, hazard ratio; CI, confidence interval. | | | | |
| **Table S16. Associations of loneliness and social isolation with subsequent risk for musculoskeletal disorders after excluding participants with missing covariates in the UKB cohort (n = 250,372).** | | | | |
|  | **N** | **Cases/Person-years** | **Model 1 HR (95% CI)** | **Model 2 HR (95% CI)** |
|  |  |  |  |  |
| **Overall MSK** | | | | |
| **Loneliness** |  |  |  |  |
| No loneliness | 237,005 | 57,119/2,824,626.4 | 1.00 Ref. | 1.00 Ref. |
| Loneliness | 13,367 | 3,748/154,379.7 | **1.25 (1.21-1.29)** | **1.14 (1.10-1.18)** |
| **Social isolation** |  |  |  |  |
| No isolated | 230,154 | 55,992/2,742,671.0 | 1.00 Ref. | 1.00 Ref. |
| Isolated | 20,218 | 4,875/236,335.0 | 1.02 (0.99-1.05) | **0.96 (0.93-0.99)** |
| **OA** | | | | |
| **Loneliness** |  |  |  |  |
| No loneliness | 237,005 | 26,247/3,024,143.8 | 1.00 Ref. | 1.00 Ref. |
| Loneliness | 13,367 | 1,664/168,304.2 | **1.20 (1.14-1.26)** | **1.09 (1.03-1.14)** |
| **Social isolation** |  |  |  |  |
| No isolated | 230,154 | 25,841/2,938,731.1 | 1.00 Ref. | 1.00 Ref. |
| Isolated | 20,218 | 2,070/253,716.9 | **0.94 (0.90-0.98)** | **0.89 (0.85-0.93)** |
| **Gout** | | | | |
| **Loneliness** |  |  |  |  |
| No loneliness | 237,005 | 2,854/3,160,548.7 | 1.00 Ref. | 1.00 Ref. |
| Loneliness | 13,367 | 199/176,901.8 | **1.30 (1.12-1.50)** | 1.06 (0.92-1.23) |
| **Social isolation** |  |  |  |  |
| No isolated | 230,154 | 2,783/3,073,614.0 | 1.00 Ref. | 1.00 Ref. |
| Isolated | 20,218 | 270/263,836.0 | 1.11 (0.98-1.26) | 0.97 (0.85-1.10) |
| **RA** | | | | |
| **Loneliness** |  |  |  |  |
| No loneliness | 237,005 | 1,652/3,164,118.0 | 1.00 Ref. | 1.00 Ref. |
| Loneliness | 13,367 | 118/177,068.5 | **1.34 (1.11-1.62)** | 1.14 (0.94-1.38) |
| **Social isolation** |  |  |  |  |
| No isolated | 230,154 | 1,601/3,077,196.0 | 1.00 Ref. | 1.00 Ref. |
| Isolated | 20,218 | 169/263,990.5 | **1.26 (1.07-1.47)** | 1.10 (0.94-1.30) |
| **Low back pain** | | | | |
| **Loneliness** |  |  |  |  |
| No loneliness | 237,005 | 4,398/3,149,622.3 | 1.00 Ref. | 1.00 Ref. |
| Loneliness | 13,367 | 393/175,624.6 | **1.65 (1.49-1.83)** | **1.38 (1.24-1.53)** |
| **Social isolation** |  |  |  |  |
| No isolated | 230,154 | 4,328/3,062,636.6 | 1.00 Ref. | 1.00 Ref. |
| Isolated | 20,218 | 463/262,610.3 | **1.26 (1.14-1.38)** | 1.08 (0.98-1.19) |
| **Neck pain** | | | | |
| **Loneliness** |  |  |  |  |
| No loneliness | 237,005 | 897/3,166,990.2 | 1.00 Ref. | 1.00 Ref. |
| Loneliness | 13,367 | 87/177,147.7 | **1.77 (1.42-2.21)** | **1.48 (1.18-1.84)** |
| **Social isolation** |  |  |  |  |
| No isolated | 230,154 | 896/3,079,732.6 | 1.00 Ref. | 1.00 Ref. |
| Isolated | 20,218 | 88/264,405.2 | 1.15 (0.93-1.44) | 0.99 (0.79-1.24) |
| Model 1: adjusted for age and sex. Model 2: additionally adjusted for assessment center, education level, current employment status, Townsend deprivation index, grip strength, systolic blood pressure, diastolic blood pressure, antihypertensive medication use, antidiabetic medication use, statin use, smoking status, alcohol consumption status, physical activity, sleep duration, healthy diet score and BMI, based on model 1. Abbreviations: MSK, musculoskeletal disorders; OA, osteoarthritis; RA, rheumatoid arthritis; HR, hazard ratio; CI, confidence interval. | | | | |

| **Table S17. Associations of loneliness and social isolation with risk for musculoskeletal disorders with additional adjustment for occupational physical workload.** | | | | |
| --- | --- | --- | --- | --- |
|  | **N** | **Cases/Person-years** | **Model 1 HR (95% CI)** | **Model 2 HR (95% CI)** |
|  |  |  |  |  |
| **Overall MSK** | | | | |
| **Loneliness** |  |  |  |  |
| No loneliness | 297,455 | 74,499/3,523,290.7 | 1.00 Ref. | 1.00 Ref. |
| Loneliness | 17,742 | 5,190/203,250.6 | **1.26 (1.22-1.29)** | **1.13 (1.10-1.17)** |
| **Social isolation** |  |  |  |  |
| No isolated | 288,539 | 72,923/3,417,926.8 | 1.00 Ref. | 1.00 Ref. |
| Isolated | 26,658 | 6,766/308,614.5 | **1.04 (1.01-1.06)** | **0.96 (0.94-0.99)** |
| **OA** | | | | |
| **Loneliness** |  |  |  |  |
| No loneliness | 297,455 | 34,434/3,782,887.7 | 1.00 Ref. | 1.00 Ref. |
| Loneliness | 17,742 | 2,320/222,150.1 | **1.21 (1.16-1.26)** | **1.08 (1.03-1.13)** |
| **Social isolation** |  |  |  |  |
| No isolated | 288,539 | 33,861/3,672,235.0 | 1.00 Ref. | 1.00 Ref. |
| Isolated | 26,658 | 2,893/332,803.0 | **0.95 (0.92-0.99)** | **0.89 (0.86-0.92)** |
| **Gout** | | | | |
| **Loneliness** |  |  |  |  |
| No loneliness | 297,455 | 3,587/3,962,484.0 | 1.00 Ref. | 1.00 Ref. |
| Loneliness | 17,742 | 277/234,281.0 | **1.37 (1.21-1.55)** | 1.13 (1.00-1.28) |
| **Social isolation** |  |  |  |  |
| No isolated | 288,539 | 3,518/3,849,666.9 | 1.00 Ref. | 1.00 Ref. |
| Isolated | 26,658 | 346/347,097.9 | 1.07 (0.96-1.19) | 0.93 (0.83-1.04) |
| **RA** | | | | |
| **Loneliness** |  |  |  |  |
| No loneliness | 297,455 | 2,262/3,966,137.8 | 1.00 Ref. | 1.00 Ref. |
| Loneliness | 17,742 | 181/234,437.9 | **1.42 (1.22-1.65)** | **1.19 (1.02-1.39)** |
| **Social isolation** |  |  |  |  |
| No isolated | 288,539 | 2,196/3,853,403.0 | 1.00 Ref. | 1.00 Ref. |
| Isolated | 26,658 | 247/347,172.7 | **1.28 (1.12-1.46)** | 1.09 (0.96-1.25) |
| **Low back pain** | | | | |
| **Loneliness** |  |  |  |  |
| No loneliness | 297,455 | 5,811/3,947,422.4 | 1.00 Ref. | 1.00 Ref. |
| Loneliness | 17,742 | 530/232,594.7 | **1.59 (1.46-1.74)** | **1.31 (1.20-1.43)** |
| **Social isolation** |  |  |  |  |
| No isolated | 288,539 | 5,729/3,834,582.0 | 1.00 Ref. | 1.00 Ref. |
| Isolated | 26,658 | 612/345,435.0 | **1.20 (1.10-1.30)** | 1.01 (0.93-1.10) |
| **Neck pain** | | | | |
| **Loneliness** |  |  |  |  |
| No loneliness | 297,455 | 1,203/3,970,414.9 | 1.00 Ref. | 1.00 Ref. |
| Loneliness | 17,742 | 110/234,692.6 | **1.58 (1.30-1.92)** | **1.29 (1.06-1.57)** |
| **Social isolation** |  |  |  |  |
| No isolated | 288,539 | 1,187/3,857,312.5 | 1.00 Ref. | 1.00 Ref. |
| Isolated | 26,658 | 126/347,795.1 | 1.19 (0.99-1.43) | 1.00 (0.83-1.21) |
| Model 1: adjusted for age and sex. Model 2: additionally adjusted for assessment center, education level, current employment status, Townsend deprivation index, grip strength, systolic blood pressure, diastolic blood pressure, antihypertensive medication use, antidiabetic medication use, statin use, smoking status, alcohol consumption status, physical activity, sleep duration, healthy diet score, BMI, and occupational physical workload based on model 1.  Abbreviations: MSK, musculoskeletal disorders; OA, osteoarthritis; RA, rheumatoid arthritis; HR, hazard ratio; CI, confidence interval.  Notes: Occupational physical workload were assessed using self-reported data on the question: “Does your work involve heavy manual or physical work?” (field ID: 816). | | | | |

| **Table S18. Associations of loneliness and social isolation with risk for musculoskeletal disorders with additional adjustment for subclinical musculoskeletal symptoms.** | | | | |
| --- | --- | --- | --- | --- |
|  | **N** | **Cases/Person-years** | **Model 1 HR (95% CI)** | **Model 2 HR (95% CI)** |
|  |  |  |  |  |
| **Overall MSK** | | | | |
| **Loneliness** |  |  |  |  |
| No loneliness | 297,455 | 74,499/3,523,290.7 | 1.00 Ref. | 1.00 Ref. |
| Loneliness | 17,742 | 5,190/203,250.6 | **1.26 (1.22-1.29)** | **1.11 (1.08-1.14)** |
| **Social isolation** |  |  |  |  |
| No isolated | 288,539 | 72,923/3,417,926.8 | 1.00 Ref. | 1.00 Ref. |
| Isolated | 26,658 | 6,766/308,614.5 | **1.04 (1.01-1.06)** | **0.96 (0.94-0.99)** |
| **OA** | | | | |
| **Loneliness** |  |  |  |  |
| No loneliness | 297,455 | 34,434/3,782,887.7 | 1.00 Ref. | 1.00 Ref. |
| Loneliness | 17,742 | 2,320/222,150.1 | **1.21 (1.16-1.26)** | **1.05 (1.00-1.09)** |
| **Social isolation** |  |  |  |  |
| No isolated | 288,539 | 33,861/3,672,235.0 | 1.00 Ref. | 1.00 Ref. |
| Isolated | 26,658 | 2,893/332,803.0 | **0.95 (0.92-0.99)** | **0.89 (0.86-0.93)** |
| **Gout** | | | | |
| **Loneliness** |  |  |  |  |
| No loneliness | 297,455 | 3,587/3,962,484.0 | 1.00 Ref. | 1.00 Ref. |
| Loneliness | 17,742 | 277/234,281.0 | **1.37 (1.21-1.55)** | 1.12 (0.99-1.27) |
| **Social isolation** |  |  |  |  |
| No isolated | 288,539 | 3,518/3,849,666.9 | 1.00 Ref. | 1.00 Ref. |
| Isolated | 26,658 | 346/347,097.9 | 1.07 (0.96-1.19) | 0.93 (0.83-1.04) |
| **RA** | | | | |
| **Loneliness** |  |  |  |  |
| No loneliness | 297,455 | 2,262/3,966,137.8 | 1.00 Ref. | 1.00 Ref. |
| Loneliness | 17,742 | 181/234,437.9 | **1.42 (1.22-1.65)** | **1.17 (1.00-1.36)** |
| **Social isolation** |  |  |  |  |
| No isolated | 288,539 | 2,196/3,853,403.0 | 1.00 Ref. | 1.00 Ref. |
| Isolated | 26,658 | 247/347,172.7 | **1.28 (1.12-1.46)** | 1.10 (0.96-1.25) |
| **Low back pain** | | | | |
| **Loneliness** |  |  |  |  |
| No loneliness | 297,455 | 5,811/3,947,422.4 | 1.00 Ref. | 1.00 Ref. |
| Loneliness | 17,742 | 530/232,594.7 | **1.59 (1.46-1.74)** | **1.27 (1.16-1.39)** |
| **Social isolation** |  |  |  |  |
| No isolated | 288,539 | 5,729/3,834,582.0 | 1.00 Ref. | 1.00 Ref. |
| Isolated | 26,658 | 612/345,435.0 | **1.20 (1.10-1.30)** | 1.02 (0.93-1.11) |
| **Neck pain** | | | | |
| **Loneliness** |  |  |  |  |
| No loneliness | 297,455 | 1,203/3,970,414.9 | 1.00 Ref. | 1.00 Ref. |
| Loneliness | 17,742 | 110/234,692.6 | **1.58 (1.30-1.92)** | **1.24 (1.02-1.52)** |
| **Social isolation** |  |  |  |  |
| No isolated | 288,539 | 1,187/3,857,312.5 | 1.00 Ref. | 1.00 Ref. |
| Isolated | 26,658 | 126/347,795.1 | 1.19 (0.99-1.43) | 1.01 (0.83-1.22) |
| Model 1: adjusted for age and sex. Model 2: additionally adjusted for assessment center, education level, current employment status, Townsend deprivation index, grip strength, systolic blood pressure, diastolic blood pressure, antihypertensive medication use, antidiabetic medication use, statin use, smoking status, alcohol consumption status, physical activity, sleep duration, healthy diet score, BMI, and subclinical musculoskeletal symptoms based on model 1.  Abbreviations: MSK, musculoskeletal disorders; OA, osteoarthritis; RA, rheumatoid arthritis; HR, hazard ratio; CI, confidence interval.  Notes: Subclinical musculoskeletal symptoms were assessed using self-reported pain in the month prior to baseline (neck/shoulder, back, hip, and knee pain; field ID: 6159). | | | | |

| **Table S19. Associations of loneliness and social isolation with subsequent risk for musculoskeletal disorders after excluding traditional risk factors.** | | | | |
| --- | --- | --- | --- | --- |
|  | **N** | **Cases/Person-years** | **Model 1 HR (95% CI)** | **Model 2 HR (95% CI)** |
|  |  |  |  |  |
| **Overall MSK** | | | | |
| **Loneliness** |  |  |  |  |
| No loneliness | 297,455 | 74,499/3,523,290.7 | 1.00 Ref. | 1.00 Ref. |
| Loneliness | 17,742 | 5,190/203,250.6 | **1.26 (1.22-1.29)** | **1.19 (1.15-1.22)** |
| **Social isolation** |  |  |  |  |
| No isolated | 288,539 | 72,923/3,417,926.8 | 1.00 Ref. | 1.00 Ref. |
| Isolated | 26,658 | 6,766/308,614.5 | **1.04 (1.01-1.06)** | 0.99 (0.96-1.01) |
| **OA** | | | | |
| **Loneliness** |  |  |  |  |
| No loneliness | 297,455 | 34,434/3,782,887.7 | 1.00 Ref. | 1.00 Ref. |
| Loneliness | 17,742 | 2,320/222,150.1 | **1.21 (1.16-1.26)** | **1.15 (1.10-1.20)** |
| **Social isolation** |  |  |  |  |
| No isolated | 288,539 | 33,861/3,672,235.0 | 1.00 Ref. | 1.00 Ref. |
| Isolated | 26,658 | 2,893/332,803.0 | **0.95 (0.92-0.99)** | **0.91 (0.88-0.95)** |
| **Gout** | | | | |
| **Loneliness** |  |  |  |  |
| No loneliness | 297,455 | 3,587/3,962,484.0 | 1.00 Ref. | 1.00 Ref. |
| Loneliness | 17,742 | 277/234,281.0 | **1.37 (1.21-1.55)** | **1.21 (1.07-1.37)** |
| **Social isolation** |  |  |  |  |
| No isolated | 288,539 | 3,518/3,849,666.9 | 1.00 Ref. | 1.00 Ref. |
| Isolated | 26,658 | 346/347,097.9 | 1.07 (0.96-1.19) | 0.95 (0.85-1.06) |
| **RA** | | | | |
| **Loneliness** |  |  |  |  |
| No loneliness | 297,455 | 2,262/3,966,137.8 | 1.00 Ref. | 1.00 Ref. |
| Loneliness | 17,742 | 181/234,437.9 | **1.42 (1.22-1.65)** | **1.28 (1.10-1.49)** |
| **Social isolation** |  |  |  |  |
| No isolated | 288,539 | 2,196/3,853,403.0 | 1.00 Ref. | 1.00 Ref. |
| Isolated | 26,658 | 247/347,172.7 | **1.28 (1.12-1.46)** | **1.16 (1.01-1.32)** |
| **Low back pain** | | | | |
| **Loneliness** |  |  |  |  |
| No loneliness | 297,455 | 5,811/3,947,422.4 | 1.00 Ref. | 1.00 Ref. |
| Loneliness | 17,742 | 530/232,594.7 | **1.59 (1.46-1.74)** | **1.43 (1.31-1.56)** |
| **Social isolation** |  |  |  |  |
| No isolated | 288,539 | 5,729/3,834,582.0 | 1.00 Ref. | 1.00 Ref. |
| Isolated | 26,658 | 612/345,435.0 | **1.20 (1.10-1.30)** | 1.08 (0.99-1.18) |
| **Neck pain** | | | | |
| **Loneliness** |  |  |  |  |
| No loneliness | 297,455 | 1,203/3,970,414.9 | 1.00 Ref. | 1.00 Ref. |
| Loneliness | 17,742 | 110/234,692.6 | **1.58 (1.30-1.92)** | **1.37 (1.13-1.67)** |
| **Social isolation** |  |  |  |  |
| No isolated | 288,539 | 1,187/3,857,312.5 | 1.00 Ref. | 1.00 Ref. |
| Isolated | 26,658 | 126/347,795.1 | 1.19 (0.99-1.43) | 1.06 (0.88-1.28) |
| Model 1: adjusted for age and sex. Model 2: additionally adjusted for assessment center, education level, current employment status, Townsend deprivation index, grip strength, systolic blood pressure, diastolic blood pressure, antihypertensive medication use, antidiabetic medication use, and statin use based on model 1. Abbreviations: MSK, musculoskeletal disorders; OA, osteoarthritis; RA, rheumatoid arthritis; HR, hazard ratio; CI, confidence interval. | | | | |

| **Table S20. Stability of social isolation and loneliness across instances subset with repeated assessments.** | | | | | |
| --- | --- | --- | --- | --- | --- |
| **Items** | | **Social Isolation** | | **Loneliness** | |
| **Pair** | **Pattern** | **N** | **%** | **N** | **%** |
| **0-1** | **Never** | 11,844 | 88.7 | 12,007 | 92.8 |
|  | **Incident** | 582 | 4.4 | 353 | 2.7 |
|  | **Remitted** | 468 | 3.5 | 332 | 2.6 |
|  | **Persistent** | 456 | 3.4 | 251 | 1.9 |
|  | **Overall** | 13,350 | 100 | 12,943 | 100 |
|  | **Agreement %** |  | 92.1 |  | 94.7 |
| **0-2** | **Never** | 43,838 | 87.9 | 44,639 | 92.7 |
|  | **Incident** | 2,632 | 5.3 | 1,420 | 2.9 |
|  | **Remitted** | 2,054 | 4.1 | 1,361 | 2.8 |
|  | **Persistent** | 1,322 | 2.7 | 743 | 1.5 |
|  | **Overall** | 49,846 | 100 | 48,163 | 100 |
|  | **Agreement %** |  | 90.6 |  | 94.2 |
| **0-3** | **Never** | 3,836 | 87.3 | 3,962 | 92.8 |
|  | **Incident** | 293 | 6.7 | 136 | 3.2 |
|  | **Remitted** | 160 | 3.6 | 106 | 2.5 |
|  | **Persistent** | 103 | 2.3 | 65 | 1.5 |
|  | **Overall** | 4,392 | 100 | 4,269 | 100 |
|  | **Agreement %** |  | 89.7 |  | 94.3 |
| Pairs 0-1, 0-2, and 0-3 are instance 1,2,3 compared to baseline. Patterns are defined as: Never: Non-exposed at both instances (0-0). Incident: Non-exposed at first instance, exposed at second (0-1). Remitted: Exposed at first instance, non-exposed at second (1-0). Persistent: Exposed at both instances (1-1). N and % are counts and percentages per pattern, with Overall as complete-case sample size per pair. Agreement % = [(Never N + Persistent N) / Overall N] * 100, reflecting the proportion of participants with consistent classification. Analyses used complete cases due to high missingness. Pair 0-3 results may be less reliable due to small sample size (N<5,000). | | | | | |

| **Table S21. Proportion and trend of social isolation and loneliness by instance.** | | | | |
| --- | --- | --- | --- | --- |
|  | **Social Isolation** | | **Loneliness** | |
| **Instance** | **N** | **Proportion(%)** | **N** | **Proportion(%)** |
| **0** | 315,197 | 8.5 | 315,197 | 5.6 |
| **1** | 13,350 | 7.8 | 12,943 | 4.7 |
| **2** | 49,846 | 7.9 | 48,163 | 4.5 |
| **3** | 4,392 | 9.0 | 4,269 | 4.7 |
|  | Note: Linear trend slope = 0.16% per instance, p = 0.63, no significant change over time. | | Note: Linear trend slope = -0.29% per instance, p = 0.24, no significant change over time. | |
| Proportion (%) = percentage of participants with exposure per instance. Linear trend slope and p-value from regression of proportions on instance number; p > 0.05 indicates no significant change over time. Instance 3 results may be less reliable due to small sample size | | | | |


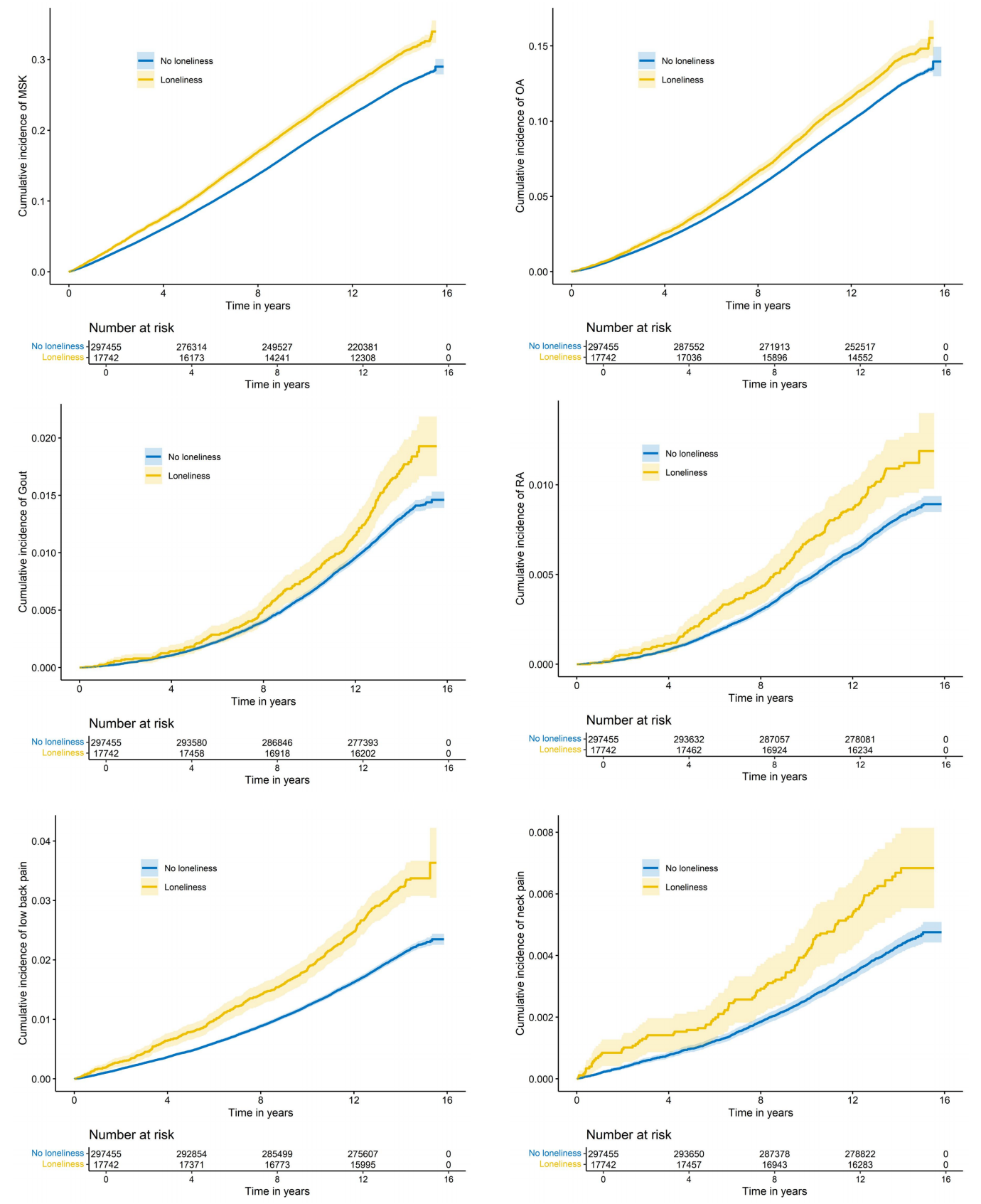


**Figure S1. KM plots for the cumulative risks of musculoskeletal disorders between groups of no-loneliness and loneliness in the UKB cohort.**
